# The Cytoplasmic Domains of *Streptococcus mutans* Membrane Protein Insertases YidC1 and YidC2 Confer Unique Structural and Functional Attributes to Each Paralog

**DOI:** 10.3389/fmicb.2021.760873

**Published:** 2021-11-02

**Authors:** Surabhi Mishra, L. Jeannine Brady

**Affiliations:** Department of Oral Biology, University of Florida, Gainesville, FL, United States

**Keywords:** structure-function, *YidC*, *Streptococcus mutans*, insertase, paralogs, bacterial stress tolerance

## Abstract

Integral and membrane-anchored proteins are pivotal to survival and virulence of the dental pathogen, *Streptococcus mutans*. The bacterial chaperone/insertase, YidC, contributes to membrane protein translocation. Unlike *Escherichia coli*, most Gram-positive bacteria contain two YidC paralogs. Herein, we evaluated structural features that functionally delineate *S. mutans* YidC1 and YidC2. Bacterial YidCs contain five transmembrane domains (TMD), two cytoplasmic loops, and a cytoplasmic tail. Because *S. mutans* YidC1 (SmYidC1) and YidC2 (SmYidC2) cytoplasmic domains (CD) are less well conserved than are TMD, we engineered ectopic expression of the 14 possible YidC1-YidC2 CD domain swap combinations. Growth and stress tolerance of each was compared to control strains ectopically expressing unmodified *yidC1* or *yidC2*. Acid and osmotic stress sensitivity are associated with *yidC2* deletion. Sensitivity to excess zinc was further identified as a Δ*yidC1* phenotype. Overall, YidC1 tolerated CD substitutions better than YidC2. Preferences toward particular CD combinations suggested potential intramolecular interactions. *In silico* analysis predicted salt-bridges between C1 and C2 loops of YidC1, and C1 loop and C-terminal tail of YidC2, respectively. Mutation of contributing residues recapitulated Δ*yidC1*- and Δ*yidC2*-associated phenotypes. Taken together, this work revealed the importance of cytoplasmic domains in distinct functional attributes of YidC1 and YidC2, and identified key residues involved in interdomain interactions.

## Introduction

The Gram-positive oral pathogen *Streptococcus mutans* is a leading cause of dental caries (Hamada and Slade, 1980), with certain strains also associated with bacterial endocarditis (Lockwood et al., 1974; McGhie et al., 1977; Moore et al., 1977; Smith et al., 1977; Vose et al., 1987; Nomura et al., 2006). This species’ ability to rapidly utilize available dietary sugars and produce large amounts of acids, coupled with high tolerance to numerous environmental stressors, gives it a competitive advantage among its co-colonizers in the oral cavity. In addition, an ability to form robust biofilms, a high level of genetic competence enabling robust adaptability, and production of anti-microbial compounds such as mutacins contribute to its pathogenic potential [reviewed in Lemos et al. (2019)]. Most known virulence attributes of *S. mutans* stem from secreted or integral membrane proteins. Thus, a more complete understanding of the components of *S. mutans* protein translocation and integral membrane insertion pathways is of significant interest.

Membrane biogenesis in bacteria is largely a co-translational process that involves the signal recognition particle (SRP) pathway working in conjunction with the SecYEG translocon and/or a membrane protein insertase called YidC [reviewed in Koch et al. (2003) and Steinberg et al. (2018)]. YidC is ubiquitously present in all three domains of life and belongs to the Oxa/Alb/YidC family of insertases found in mitochondria, chloroplasts, and bacteria, respectively. Most details related to membrane biogenesis in bacteria, including contributions by YidC, have come from research in the model Gram-negative bacterium *Escherichia coli* (Samuelson et al., 2000; Scotti et al., 2000; Urbanus et al., 2001; van Bloois et al., 2004; van der Laan et al., 2004; Yi et al., 2004; Price et al., 2010; Kumazaki et al., 2014b). However, Gram-positive bacteria differ from their Gram-negative counterparts both in terms of the composition of the translocation machinery and the essentiality of individual components for survival [reviewed in Lewis and Brady (2015)]. Three key distinctions that differentiate the protein translocation machinery of Gram-positive bacteria from Gram-negative bacteria include: 1) the non-essentiality of the SRP components, Ffh, scRNA, and/or the SRP receptor FtsY, 2) the presence of two or sometimes more paralogs of YidC in Gram-positive bacteria that can be eliminated individually without lethality, and 3) the presence of an accessory SRP protein, YlxM (Hasona et al., 2005; Williams et al., 2014). Phenotypic characterization of *S. mutans* mutants has revealed overlapping features of SRP- and YidC2-deficient strains, with both being growth impaired under environmental stress (acid/osmotic/oxidative) as well as non-stress conditions. In contrast, a Δ*yidC1* mutant has previously shown no obvious growth or stress-sensitivity phenotype (Hasona et al., 2005; Palmer et al., 2012). Combinatorial deletion of *ffh* and *yidC2*, or *yidC1* and *yidC2*, is lethal in *S. mutans*, while a Δ*ffh*/Δ*yidC1* double deletion strain survives suggesting that the presence of YidC2 alone can confer viability in this organism (Mishra et al., 2019). Membrane proteomic analyses of Δ*ffh*, Δ*yidC1*, Δ*yidC2*, and Δ*ffh*/*yidC1* strains suggested a high degree of functional redundancy between YidC1 and YidC2, with gain of function mutations within *yidC1* in a Δ*yidC2* background also identified (Mishra et al., 2019). A gain of function mutation has also been identified in *Bacillus subtilis* YidC1 (SpoIIIJ) enabling it to survive lethality caused by high σ^M^ levels that interfere with intrinsic resistance to cell wall targeting antibiotics (Zhao et al., 2019). Work to characterize *S. mutans* YidC1 and YidC2 (SmYidC1 and SmYidC2) interactomes *via* immunocapture and chemical crosslinking experiments revealed several differences between apparent YidC1 and YidC2 interaction partners, and suggested both overlapping and individual substrate preferences (Lara Vasquez et al., 2021). Taken together results of phenotypic, proteomic, and protein-protein interaction analyses of WT, Δ*yidC1*, and Δ*yidC2* mutants have suggested significant unique features of SmYidC1 and SmYidC2 despite a high degree of functional overlap. Phenotypic distinctions between *B. subtilis ΔyidC1*(*spoIIIJ)* and Δ*yidC2(yqjG)* mutants have also been reported (Errington et al., 1992; Saller et al., 2011; Zhao et al., 2019). Therefore, it is of broad general interest to understand why Gram-positive bacteria generally contain two distinct paralogs of YidC, and to identify specific structural features that confer individual activities of YidC1 compared to YidC2.

Primary sequence comparison demonstrates ∼27% identity and ∼47% homology (Emboss-Needleman tool) between *S. mutans* YidC1 and YidC2. A key feature delineating YidC1 and YidC2 is the longer and more basic C-terminal tail of YidC2. Indeed, acid stress tolerance was partially restored in a Δ*yidC2* background when the YidC1 tail was replaced with that of YidC2 (Palmer et al., 2012). Later, crystal structure characterization combined with site-directed mutagenesis of *B. halodurans* YidC2 showed that its C1 cytoplasmic loop is flexible, and that its deletion impairs membrane insertase activity (Kumazaki et al., 2014a). Primary sequence analyses and crystal structures illustrate that all mature YidCs consist of five transmembrane domains (TM2-TM6) that are interspersed with cytoplasmic loop 1 (C1), cytoplasmic loop 2 (C2), and a cytoplasmic C-terminal tail (T). In consideration of the fact that protein synthesis occurs in the cytoplasm, YidC cytoplasmic domains would be expected to participate not only in specific interactions with other co-translational translocation machinery components but also with substrates destined for insertion. In *E.* coli, incoming substrates have been hypothesized to interact initially with the YidC C1 loop followed by interaction with a conserved arginine residue within transmembrane domain TM2 for insertion (Kol et al., 2008; Kumazaki et al., 2014a,c). More recently, molecular dynamics simulation showed that the crystallographically unresolved cytoplasmic C2 loop of BhYidC2 plays an important role in stabilizing this insertase’s structure (Harkey et al., 2019).

In the current work, we engineered a panel of chimeric *yidC1/2* constructs in which one or more YidC1 cytoplasmic domains were substituted for those of YidC2, and vice versa. The chimeric variants were integrated into the bacterial chromosome and ectopically expressed from the *gtfA* locus in a genetic background in which endogenous *yidC1* and *yidC2* had both been eliminated. Each strain was characterized in terms of growth and survival under non-stress and stress conditions including acid, osmotic, and metal excess. This revealed a novel phenotype for the Δ*yidC1* mutant, that was not shared by the Δ*yidC2* strain, namely sensitivity to excess Zn(II). Three dimensional (3D)-structure prediction tools were also utilized to evaluate structural constraints within each of the various YidC1/2 chimeric proteins compared to unmodified YidC1 and YidC2. Our experimental results suggest considerable plasticity of YidC1 function in that this paralog could accommodate any combination of cytoplasmic domain substitutions without significant impact on the phenotypes tested. In contrast, YidC2 structure appeared less malleable with a preference for specific combinations of cytoplasmic domains in order to support growth under non-stress as well as environmental stress conditions. Lastly, prediction tools identified putative interactions between cytoplasmic loops 1 and 2 of YidC1, and between cytoplasmic loop 1 and the C-terminal tail of YidC2, which appeared to stem from stabilizing salt-bridges within YidC1 and YidC2, respectively. Amino acids involved in these putative salt-bridges, K91 and E190 in YidC1, and E92 and K253 in YidC2, were substituted with alanine by site-directed mutagenesis and confirmed to contribute to paralog-specific functions.

## Materials and Methods

### Bacterial Strains, Plasmids and Growth Conditions

Bacterial strains and plasmids used in this study are listed in Supplementary Table 1. All of the *S. mutans* mutants used in this study were derived from *S. mutans* strain UA159 (Ajdic et al., 2002). *S. mutans* strains were routinely grown in Todd-Hewitt broth (BBL, Becton Dickinson) supplemented with 0.3% yeast extract (THYE) at 37°C in 5% CO_2_/95% air atmosphere (v/v). Spectinomycin (1 mg ml^–1^), kanamycin (1 mg ml^–1^), or erythromycin (10 mg ml^–1^) were added, where appropriate, for the growth and selection of *S. mutans* strains. *E. coli* strain C2987 (NEB) was used for standard cloning procedures and routinely grown in Luria–Bertani medium (10 g l^–1^ tryptone, 5 g l^–1^ yeast extract and 5 g l^–1^ NaCl) at 37°C with vigorous shaking or on LB agar (1.5%) with appropriate selection. Ampicillin (100 μg ml^–1^), kanamycin (50 μg ml^–1^), or erythromycin (250 μg ml^–1^) were used for the growth and selection of *E. coli* transformants.

### Construction of Chimeric *yidC1* and *yidC2* Genes

The Gibson assembly method was used to construct 14 different chimeric genes of *yidC1* and *yidC2* with DNA encoding one, two, or three cytoplasmic domains swapped with the corresponding regions from the other paralog. Details of the chimeric YidC1/2 proteins are provided in Supplementary Table 2. DNA fragments corresponding to the specific regions of the *yidC1* and *yidC2* within each chimera were PCR-amplified using primers listed in Supplementary Table 3. PCR products were assembled between *Nde*I/*Bam*HI sites of pET15b using NEBuilder HiFi DNA Assembly kit (NEB) following the manufacturer’s instructions and transformed into C2987 competent *E. coli* (NEB) by heat shock. Chimeric *yidC1/2* fragments subcloned in pET15b were PCR-amplified using end primers and subcloned between *Xba*I/*Bsr*GI sites of the *S. mutans* integration vector, pBGE. These constructs were subsequently used for integration at the non-essential *gtfA* locus.

### Site-Directed Mutagenesis of *yidC1* and *yidC2*

A standard PCR-based technique involving partially overlapping primers (Supplementary Table 3) was used for site-directed mutagenesis. pSM15 and pSM20 were used as templates for the mutagenesis of *yidC1* and *yidC2* genes, respectively (Zheng et al., 2004). For subsequent integration into *S. mutans*, genes containing the engineered point mutations were first subcloned into the integration vector, pBGE, and transformants were selected for resistance to erythromycin.

### Construction of *Streptococcus mutans* Strains Expressing Chimeric *yidC1/2*

Construction of *S. mutans* strains expressing a single chimeric CD-substituted *yidC1* or *yidC2* backbone gene was done in three steps. First *yidC1* or *yidC2* was deleted from the UA159 genome. *yidC1* was eliminated by allelic exchange with a spectinomycin resistant marker (*aad9*) using the pCR2.1-DyidC1Sp plasmid (Palmer et al., 2012). The resultant Δ*yidC1* strain (SM2001) was next transformed with pBGE plasmids containing either the wild-type (WT) or a chimeric *yidC1* gene and selected for resistance to spectinomycin (1 mg ml^–1^) or erythromycin (10 mg ml^–1^). Lastly *yidC2* was deleted from the WT- or chimeric *yidC1*-expressing strain by allelic replacement with *aphA-3* encoding kanamycin resistance. The Δ*yidC2:aphA-3* cassette for *yidC2* deletion was generated by allelic exchange with a kanamycin resistant marker using pAH342 plasmid (Hasona et al., 2005). Similarly, chimeric *yidC2* expressing strains were constructed by initial deletion of *yidC2* using the Δ*yidC2:aphA-3* cassette followed by integration of chimeric *yidC2* at the *gtfA* locus and subsequent deletion of *yidC1* by allelic replacement with *aad9*. Expression of site-directed point mutants of *yidC1* and *yidC2* genes was carried out as described above for chimeric *yidC1* and *yidC2*. Deletion of *yidC1* and *yidC2*, and integration of point mutated or chimeric *yidC1/2*, were confirmed by PCR and sequencing of the relevant genomic loci. Production of the WT or chimeric YidC1/YidC2 polypeptide was confirmed in each genetically engineered strain by Western blot using monospecific anti-YidC1 and anti-YidC2 antibodies (Palmer et al., 2012; Supplementary Figure 1).

### Bacterial Growth Under Non-stress and Stress Conditions

Growth of each *S. mutans* strain was monitored using a Bioscreen C instrument (Growth Curves United States, Piscataway, NJ, United States). Overnight (O/N) cultures were grown in THYE with appropriate antibiotic selection at 37°C in air with 5% CO_2_. For precultures, O/N cultures were diluted 1:50 (or 1:20 for very slow growing strains) into 2 ml THYE without selection and grown to OD_600_ of 0.2–0.4. Precultures were then diluted to OD_600_ = 0.005 in 1 mL of prewarmed THYE and 300 ml of each dilution was transferred in triplicate to wells of a Bioscreen micro-well plate. A sterile mineral oil overlay (50 μL) was added on top of cultures in each well to measure growth under anaerobic conditions. Cell density was measured spectrophotometrically (OD_600_) every 30 min for up to 48 h of growth at 37°C. To assess the effect of oxygen stress, the overlay of sterile mineral oil was omitted. To assess the effect of acid stress, cells were grown in THYE adjusted to pH 5.0. To assess osmotic stress, THYE was supplemented with 3% NaCl. Metal intoxication experiments involved addition of 5 mM FeSO_4_ (added from a stock of 1 M FeSO_4_ prepared in 0.1 N HCl), 5 mM MnCl_2_, or 2.5 mM ZnCl_2_ to THYE. Bioscreen plate wells were not overlayed with mineral oil when bacteria were grown under metal-excess conditions.

### Measurement of Plating Efficiency by Spot Dilution

Strains were grown to mid-exponential phase (OD_600_ = 0.2–0.4) in THYE from an O/N culture in THYE with appropriate antibiotic selection. Cultures were then adjusted to OD_600_ = 0.2, and serial 10-fold dilutions were made in THYE. Four microliter aliquots of each dilution were spotted on THYE agar (pH 7.0, pH 5.0, 3% NaCl, 5 mM FeSO_4_, 5 mM MnCl_2_, or 2.5 mM ZnCl_2_). Plates were incubated at 37°C in air with 5% CO_2_ for 2 days before visual inspection and documentation.

### Bioinformatic Analyses and 3D Structure Prediction and Comparison

YidC sequence logo was created by alignment of YidC1 and YidC2 sequences of ten Gram-positive bacterial species using the weblogo tool^1^ (Schneider and Stephens, 1990). The clustal omega alignment of YidC1 and YidC2 sequences was entered into the webtool to generate the sequence logo image. Physico-chemical properties of various chimeric YidC1 and YidC2 proteins were evaluated with the Protparam web tool^2^ using their primary amino acid sequences (Gasteiger et al., 2005). Three-dimensional structures of the wild-type proteins and chimeric YidC1 and YidC2 variants were predicted using I-TASSER (*Roy et al., 2010*). Protein structures were analyzed with Pymol (PyMOL Molecular Graphics System, Version 1.2r3pre, Schrödinger, LLC). Structure comparisons were done to determine the best alignment (lowest root mean square deviation over the largest number of amino acids) of two proteins using the cealign plugin in Pymol (Shindyalov and Bourne, 1998).

## Results

### Cytoplasmic Domains of YidC1 and YidC2 Are Less Conserved Than Their *Trans-*Membrane Domains

Functional differences in paralogous proteins often arise as a result of sequence-structure differences acquired through mutations (Conrad and Antonarakis, 2007), with other contributing factors including distinct gene neighborhood, operon organization, and genetic regulation (Zallot et al., 2016). A high resolution crystal structure of BhYidC2 lacking the N-terminal domain and C-terminal tail region is available (Kumazaki et al., 2014a); however, this information alone is not sufficient to explain distinct functional attributes of multiple YidC paralogs of Gram-positive bacteria.

**FIGURE 1:**
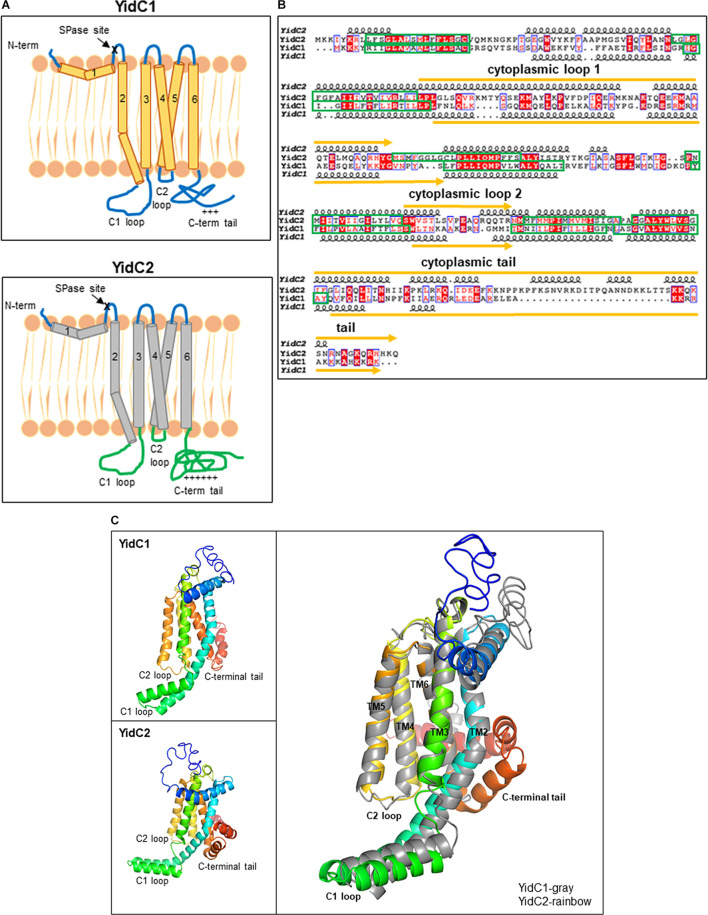
*In silico* comparison of *S. mutans* YidC1 and YidC2. **(A)** Predicted membrane topologies of YidC1 and YidC2 using TM-Pred [TMpred Server (vital-it.ch)]. The mature YidC1 and YidC2 proteins each contain five TM domains and three cytoplasmic regions: C1 loop, C2 loop, and C-terminal tail. “ + + + ” or “ + + + + + ” refers to the relative positivity of the charged C-terminal tails of Sm YidC1 and YidC2. **(B)** Alignment of primary and secondary structures of SmYidC1 and SmYidC2 predicted by I-TASSER [I-TASSER server for protein structure and function prediction (umich.edu)]. Amino acid sequences were aligned using the Clustal Omega Program (EMBOSS-Needle) and presented using the ESPript2.2 program (Gouet et al., 1999). Identical residues are highlighted by red boxes, while similar residues are highlighted by red text. Indicated secondary structural helices are based on iTasser-predicted 3D structures. Green boxes correspond to transmembrane domains and yellow lines represent cytoplasmic domains. **(C)** Cartoon representations of the superimposed I-TASSER-predicted 3D structures of YidC1 and YidC2 with YidC1 shown in gray and YidC2 shown as rainbow colored from N- to C-terminus. The YidC2 structure superimposes on YidC1 with a root-mean-square deviation of 4.11 Å for all the Cα atoms (for over 232 residues).

To begin to explore the structural features that likely contribute to characteristic functional activities of SmYidC1 vs. SmYidC2, we first carried out *in silico* analysis and comparison of their structures. Pairwise comparison showed ∼27% identity and ∼47% similarity between SmYidC1 and SmYidC2 (EMBOSS Needle tool). Both paralogs are predicted to be lipoproteins with cleavable signal peptide sequences (PRED-LIPO and Signal P 4.0), and hydropathy analysis by various webtools indicates a post-cleavage structure containing five transmembrane domains (TMDs) connected by two cytoplasmic loops: C1 and C2, and a hydrophilic C-terminal tail for both proteins (Stoffel, 1993; Krogh et al., 2001; Bagos et al., 2008; Petersen et al., 2011; Figure 1A). Despite low sequence similarity, SmYidC1 and SmYidC2 are predicted to have similar topological architectures except that the cytoplasmic C-terminal tail region of YidC2 is significantly longer and more basic than that of YidC1 (63 compared to 38 amino acids). Overlaying topology predictions onto the aligned amino acid sequences illustrates that a greater degree of identity/homology (red boxes/texts in Figure 1B, respectively) is observed between the TMDs of SmYidC1 and SmYidC2 compared to their cytoplasmic loops and C-terminal tails (Figure 1B).

We also extended our pairwise comparison of SmYidC1 and SmYidC2 to the respective YidC orthologs from other Gram-positive bacteria to determine if the sequence diversity observed within the cytoplasmic regions is limited to *S. mutans* or extends to other species. The results of multiple YidC1/YidC2 sequence alignments from various Gram-positive bacteria are shown in Supplementary Figure 2. Conservation of residues was plotted using Weblogo (Supplementary Figure 3). Interestingly, in all cases of dual YidC paralogs, higher sequence conservation was observed within regions that align with the SmYidC1 and SmYidC2 TMDs, especially TM3. The highest degree of variation was observed among the YidC C-terminal tails, which did not align significantly enough to be included in the Weblogo representation (Supplementary Figures 2, 3).

Since crystallographic data is not available for SmYidC1/2, we used I-TASSER (Iterative Threading ASSEmbly Refinement) to generate their 3D-models (Roy et al., 2010; Figure 1C). Three dimensional models of SmYidC1 and SmYidC2 resemble the crystal structure of the BhYidC (PDB nos. 3WO6 and 3WO7) core region, which also contains TMDs (TM2-TM5) and three cytoplasmic domains: C1 loop, C2 loop, and C-terminal tail. When predicted 3D-models of SmYidC1 and SmYidC2 were compared to one another, the Cα atoms superimposed with a root mean square deviation (RMSD) of 4.11 Å over 232 residues (Figure 1C). The 3D structure comparison further showed that the C1 and C2 cytoplasmic loops of YidC1 and YidC2, as well as their C-terminal tails, did not superimpose as well as their TMDs. Taken together, sequence and structural analyses clearly indicate that the TMDs are more highly conserved than are the cytoplasmic domains of YidC1 and YidC2. We therefore hypothesized that cytoplasmic regions likely confer paralog-specific functional activities to the two *S. mutans* insertases.

### Design and *in silico* Characterization of Chimeric *yidC1/2*

**FIGURE 2:**
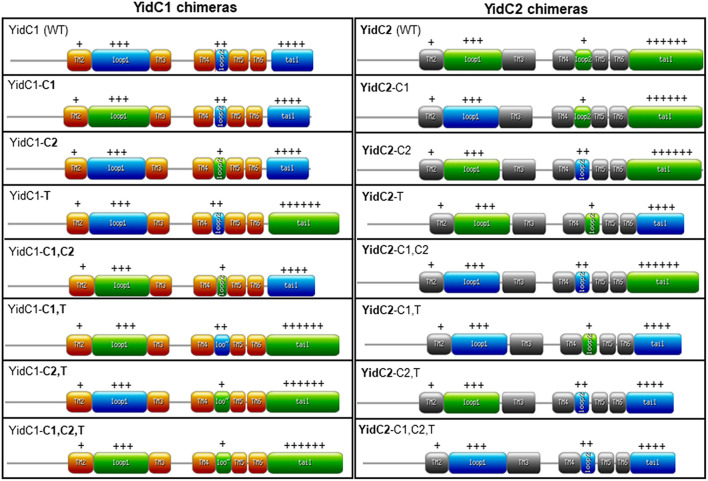
Schematic representation of unmodified and chimeric YidC1 and YidC2 polypeptides. YidC1 transmembrane (TM) domains are illustrated in orange and cytoplasmic domains (CD) in blue. YidC2 TM domains are illustrated in gray and CD in green. Chimeric constructs were designed using the web-based https://prosite.expasy.org/mydomains/tool. Regular text indicates YidC1-derived backbone or CD. Bold text indicates YidC2-derived backbone or CD. “ + + + ” or “ + + + + + ” refers to the relative positivity of the charged C-terminal tails of Sm YidC1 and YidC2.

Previous work from our laboratory revealed the significance of the C-terminal tails in conferring functional differences between SmYidC1 and SmYidC2. When a Δ*yidC2* deletion strain was engineered to harbor tail-swapped versions of YidC1 or YidC2, stress-sensitivity of the mutant strain was partially restored by ectopic expression of chimeric YidC1 harboring the YidC2 tail, while the mutant phenotype was worsened by expression of chimeric YidC2 harboring the YidC1 tail (Palmer et al., 2012). Notably, these experiments were conducted in a Δ*yidC2* mutant background, in which a functionally active native YidC1 remained in addition to the chimeric ectopic YidCs. In the current work, we extended our analysis by swapping all three cytoplasmic domains including loops C1, C2, and the C-terminal tails singly and in combination, and constitutively expressing each chimeric gene as the sole paralog from the ectopic *gtfA* locus in the genome so that the contribution of each individual domain toward the particular phenotype of interest could be specifically addressed. Combinatorial exchange of two and three different cytoplasmic domains were included in these experiments so that potential interactions among the YidC1 or YidC2 cytoplasmic domains could also be assessed. Schematic representations of YidC1, YidC2, and the 14 different domain swapped chimeric constructs are illustrated in Figure 2. Throughout the paper, the YidC1 backbone and cytoplasmic domains are indicated in regular type, while the YidC2 backbone and cytoplasmic domains are indicated in bold.

*In silico* characterization of YidC1, YidC2, and their chimeric variants included determination of pI, number of positively and negatively charged amino acids, instability index (II), grand average hydropathy (GRAVY) using Exapsy ProtParam tools and structural comparison using predicted 3D models (Table 1 and Supplementary Figure 4). Theoretical pI calculations showed that both YidC1 and YidC2 are basic proteins (pI = 10 and 10.25, respectively), with the pI values of chimeric YidC1 and YidC2 proteins remaining close to those of the WT versions. Next, we determined the hydrophobicity of the proteins by calculating their GRAVY scores such that positive values represent hydrophobic proteins while negative values indicate hydrophilic proteins (Kyte and Doolittle, 1982). As expected for membrane proteins, GRAVY scores of YidC1 and YidC2 were both above 0; however, the C1 loop of YidC1 and C-terminal tail of YidC2 were more hydrophilic compared to their respective counterparts. Swapping these domains indeed lowered the predicted hydrophobicity of the chimeric proteins.

**TABLE 1:** Physico-chemical evaluation of YidC1 and YidC2 chimeric polypeptides.

Proteins	No. of amino acids	Theoretical pI	Total no. of charged residues	Molecular weight (kD)	GRAVY	Instability Index	RMSD (YidC1, αC-αC)	RMSD (YidC2, αC-αC)
YidC1	271	10	+ 40 and −21	31.327	0.093	43.53	NA	4.12 Å
**YidC2**	310	10.25	+ 41 and −13	34.923	0.023	38.36	4.12 Å	NA

**Chimeric YidCs**								

YidC1-**C1**	273	10.05	+ 39 and −20	31.588	0.178	46.32	3.73 Å	3.16 Å
YidC1-**C2**	273	10	+ 39 and −21	31.596	0.052	45.82	3.65 Å	3.23 Å
YidC1-**T**	297	10.08	+ 44 and −20	34.241	–0.094	41.57	3.29 Å	3.2 Å
YidC1-**C1, C2**	275	10.05	+ 38 and −20	31.857	0.136	48.57	3.97 Å	3.65 Å
YidC1-**C1, T**	299	10.13	+ 43 and −19	34.503	–0.016	44.13	3.19 Å	3.63 Å
YidC1-**C2, T**	299	10.09	+ 43 and −20	34.510	–0.131	43.67	4.13 Å	2.76 Å
YidC1-**C1, C2, T**	301	10.14	+ 42 and −19	34.772	–0.053	46.21	4.2 Å	4.43 Å
**YidC2**-C1	308	10.19	+ 42 and −14	34.662	–0.053	36.13	3.89 Å	3.34 Å
**YidC2**-C2	308	10.24	+ 42 and −13	34.654	0.059	25.9	2.93 Å	2.85 Å
**YidC2**-T	284	10.18	+ 37 and −14	32.009	0.212	39.94	4.02 Å	2.80 Å
**YidC2**-C1, C2	306	10.18	+ 43 and −14	34.393	–0.017	33.64	3.94 Å	3.4 Å
**YidC2**-C1, T	282	10.12	+ 38 and −15	31.748	0.131	37.51	3.6 Å	2.46 Å
**YidC2**-C2, T	282	10.17	+ 38 and −14	31.740	0.254	37.26	3.95 Å	2.13 Å
**YidC2**-C1, C2, T	280	10.12	+ 39 and −15	31.478	0.172	34.79	4.4 Å	2.82 Å

**Point mutations**								

YidC1^*K91A*^	271	9.97	+ 39 and −21	31.27	0.114	43.53	3.50 Å	2.68 Å
YidC1^*E190A*^	271	10.05	+ 40 and −20	31.27	0.113	42.82	3.50 Å	3.53 Å
YidC2^*E92A*^	310	10.29	+ 41 and −12	34.86	0.04	38.33	3.02 Å	2.82 Å
YidC2^*K253A*^	310	10.23	+ 40 and −13	34.86	0.041	36.41	3.61 Å	2.88 Å

*YidC1 backbone and cytoplasmic domains are indicated in plain text while YidC2 backbone and cytoplasmic domains are indicated in bold.*

We were also interested to know the effect of individual and combinatorial domain substitutions on chimeric YidC1/2 structures (Supplementary Figure 4). We used I-TASSER to predict the 3D structures of each variant and compared them with the 3D structures of the WT proteins, evaluating similarity with each WT protein based on lowest RMSD values over the largest number of amino acids using the cealign plugin in Pymol (Shindyalov and Bourne, 1998; Table 1). Alignment data showed higher RMSD values for most of the YidC1 chimeras, which suggests that YidC1 structure is less rigid compared to that of YidC2. That is, most of the domain swaps impacted the 3D structure of YidC1. On the other hand, most of the chimeric YidC2 proteins, except **YidC2-**C1 and **YidC2-**C1,C2, aligned better with unmodified YidC2 than with unmodified YidC1. Therefore, the core domain structure of YidC2 appears more rigid with most of the domain swaps not substantially deviating from that predicted for unmodified YidC2. In agreement with physicochemical characterization of the chimeric YidC proteins, C1 loop substitutions in both YidC1 and YidC2 notably altered alignment with their respective unmodified proteins. Substitution of all three domains within YidC1 (YidC1-**C1,C2,T**) made this chimera dissimilar to both YidC1 and YidC2, while **YidC2-**C1,C2,T was similar to YidC2 but did not align well with YidC1.

### Construction and Characterization of *yidC1* and *yidC2* Ectopic Expression Strains

We next evaluated *S. mutans* strains expressing the 14 different chimeric versions of *yidC1* or *yidC2*. The chimeric *yidC* genes, or unmodified controls, were introduced into the non-essential *gtfA* locus for expression in a *S. mutans* genetic background in which native *yidC1* and *yidC2* had been both eliminated. Strain constructions were carried out in a step-wise manner to overcome the lethality caused by simultaneous elimination of genes encoding both YidCs. Thus, native *yidC1* or *yidC2* was deleted before integrating a chimeric or unmodified gene at the *gtfA* locus. This was followed by deletion of the residual native *yidC2* or *yidC1*, and resulted in each engineered *S. mutans* strain ectopically expressing a single *yidC* variant under control of the *gtfA* promoter. Production of each WT or chimeric YidC1/2 polypetide was confirmed by Western blot with appropriate monospecific anti-YidC1 or YidC2 rabbit antisera generated against synthetic peptides corresponding to the cytoplasmic tail domains (Supplementary Figure 1).

**FIGURE 3:**
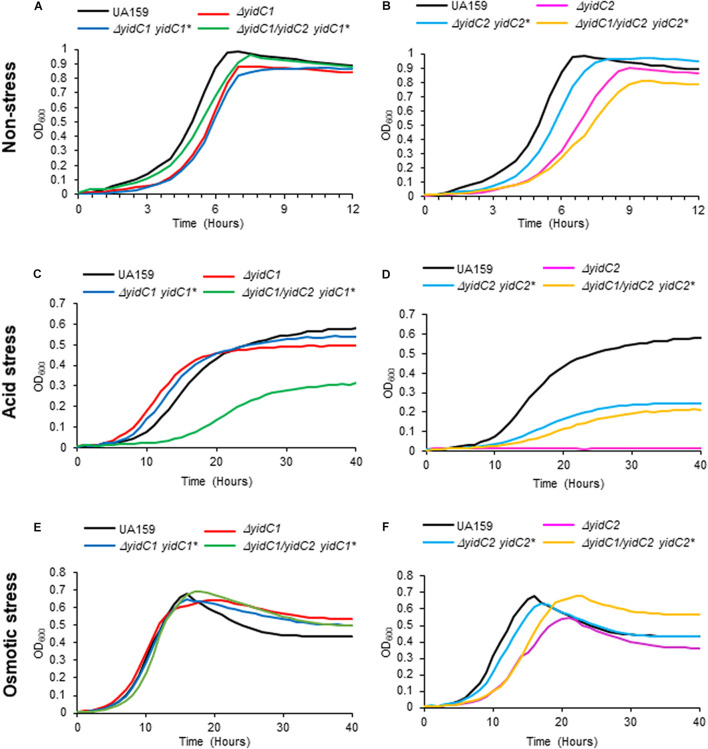
Evaluation of growth of *S. mutans* wild-type, D*yidC1*/*2* mutants, and ectopic expression strains under non-stress, acid-stress, and salt-stress conditions. Growth of the wild-type UA159 parent strain compared to Δ*yidC1* **(A,C,E)** or Δ*yidC2* **(B,D,F)** deletion mutants, and strains complemented by ectopic expression (*) from the *gtfA* locus of *yidC1* or *yidC2* in single Δ*yidC1/2* or double Δ*yidC1*/Δ*yidC2* backgrounds. **(A,B)** Growth in THYE, pH 7). **(C,D)** Growth in THYE acidified to pH 5.0. **(E,F)** Growth in THYE supplemented with 3% NaCl. Growth measurements were carried out at least three times with each sample assayed in triplicate. Data shown are mean replicates of an independent experiment.

After their construction, we first evaluated the growth of the *S. mutans* strains that ectopically expressed *yidC1* or *yidC2* in both single deletion and double Δ*yidC1*/Δ*yidC2* deletion backgrounds compared to the UA159 parental strain under non-stress and environmental stress conditions (acid and osmotic) (Figure 3). As expected, growth under all three conditions was notably diminished only in the *yidC2* single mutant. When growth was evaluated under aerated conditions in both broth culture and on agar plates, results were similar to those under non-stress conditions (Supplementary Figure 5). Under non-stress and osmotic stress conditions, the Δ*yidC1*/Δ*yidC2*-*yidC1*^∗^ ectopic expression strain grew at a rate comparable to that of the UA159 parent, while the Δ*yidC1*/Δ*yidC2*-*yidC2*^∗^ ectopic expression strain grew more slowly (Figures 3A,B,E,F and Supplementary Table 4). Neither ectopic expression of *yidC1*, nor *yidC2*, restored the WT level of growth under acid stress conditions (Figures 3C,D). Incomplete rescue when *yidC1* or *yidC2* were expressed ectopically rather than from their endogenous locations likely reflects the lack of native gene regulation when non-native promoters and loci are employed. Indeed, our prior membrane proteome characterization of WT, Δ*yidC1*, and Δ*yidC2* strains showed that YidC2 levels were increased in a Δ*yidC1* background, although a similar increase in YidC1 was not observed in the Δ*yidC2* mutant (Mishra et al., 2019). Because the focus of the current work is a structure-function analysis of the YidC1 and YidC2 polypeptides themselves, and not an evaluation of gene regulation or expression, all subsequent functional assays were performed using the ectopically-expressed unmodified *yidC1* or *yidC2* genes in a Δ*yidC1*/Δ*yidC2* background, rather than the UA159 parent strain, as the basis for of comparison of the chimeric *yidC1/2* variants.

### YidC1, but Not YidC2, Successfully Tolerates Individual or Combinatorial Substitution of Its Cytoplasmic Domains

**FIGURE 4:**
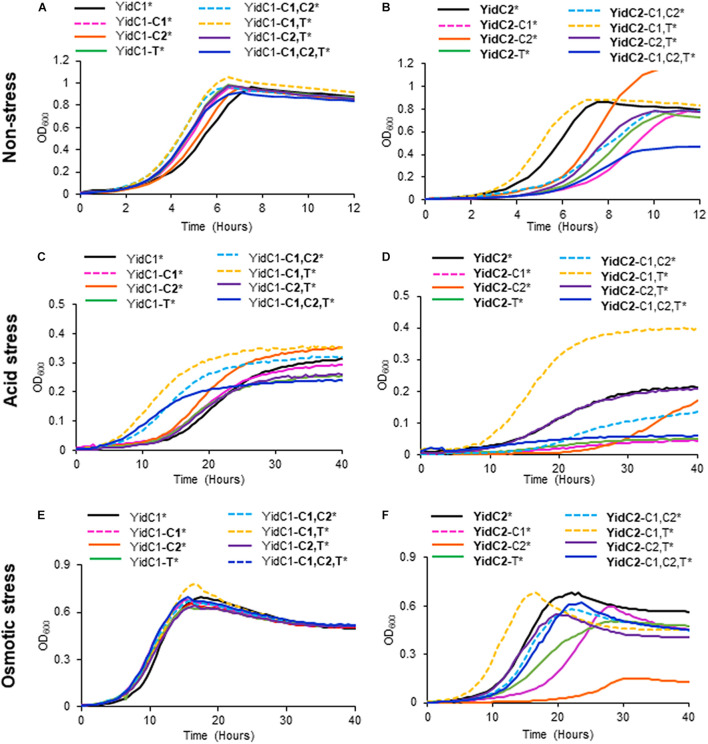
Effect of YidC1/2 cytoplasmic domain substitutions on *S. mutans* growth under non-stress, acid-stress, or salt-stress conditions. Growth of strains complemented by ectopic expression (*) from the *gtfA* locus of unmodified *yidC1* **(A,C,E)** or *yidC2* **(B,D,F)** compared to corresponding cytoplasmic domain (CD) swap variants in a double Δ*yidC1*/Δ*yidC2* background. **(A,B)** Growth in THYE, pH 7). **(C,D)** Growth in THYE acidified to pH 5.0. **(E,F)** Growth in THYE supplemented with 3% NaCl. Growth measurements were carried out at least three times with each sample assayed in triplicate. Data shown are mean replicates of an independent experiment. Regular text indicates YidC1 backbone or CD. Bold text indicates YidC2 backbone or CD.

None of the individual or combinatorial substitutions of YidC2 cytoplasmic domains into YidC1 resulted in diminished growth of *S. mutans* compared to ectopic expression of unmodified *yidC1* under non-stress conditions (Figure 4A and Supplementary Table 4). Similar results were obtained when each of the strains was grown on solid media (Supplementary Figure 6). These results suggest that any of the three cytoplasmic domains of YidC2 can successfully replace those of YidC1, individually or in combination, to support growth and survival of *S. mutans* in the absence of external environmental stressors. Next, we tested strains harboring chimeric YidC2 variants compared to the unmodified YidC2 polypeptide (Figure 4B). Most of the chimeric YidC2 variants, except for **YidC2**-C1,T, exhibited decreased growth compared to the unmodified YidC2 control strain. Similar results were observed when the strains were grown under aerated rather than anaerobic conditions except that the **YidC2**-C1,T variant more closely resembled that of the unmodified YidC2 control (Supplementary Figure 6 and Supplementary Table 4). Growth on solid media also followed a similar pattern to that observed in liquid media (Supplementary Figure 6). The strain harboring the **YidC2-**C2 chimera was noteworthy in that after an extended lag phase it achieved a substantially higher yield compared to the unmodified YidC2 control strain (Figure 4B). Subsequent subculture in THYE did not alter this strain’s growth kinetics suggesting that delayed growth and increased yield was not the result of a suppressor mutation, but rather physiological adaptation in broth culture. The negative impact of expression of most of the chimeric *yidC2* variants on *S. mutans* growth illustrates that individual YidC1 and YidC2 cytoplasmic domains are not functionally equivalent. YidC2 structure appears less malleable and tolerant of substitutions than that of YidC1. YidC2 function was perturbed by all domain substitutions except for **YidC2**-C1,T. These results are consistent with the notion that putative interactions occur between cytoplasmic domains such that specific combinatorial substitutions are preferred. The C1 loops of YidC1 and YidC2 are of similar size suggesting that primary sequence rather than overall length plays an important role in C1 functional attributes. In contrast, the C2 loops and C-terminal tails of YidC1 and YidC2 vary in both size and sequence.

### Domain Substitutions Within YidC1 Impact *S. mutans* Tolerance to Acid and Osmotic Stress Less So Than Domain Substitutions Within YidC2

Growth in acidified medium (pH 5.0) of the strain with chimeric YidC1-**T** was negatively affected, whereas the strain harboring YidC1-**C2** demonstrated improved growth, and that with YidC1-**C1** appeared unaffected (Figure 4C and Supplementary Table 4). This suggests specific influences of the cytoplasmic tail and C2 loop on the structure and function of YidC1 compared to YidC2. Combinatorial replacement of YidC2 C1 and C2, or the YidC2 C1 and tail domains, into YidC1 revealed notably enhanced growth of the chimeric strains in acid conditions (Figure 4C and Supplementary Table 4). This suggests potential interactions between certain pairs of cytoplasmic domains that may not be replicated with heterologous partners. Surprisingly, growth yield under acid conditions was notably diminished in the YidC1-**C1,C2,T** triple chimeric strain. This pronounced negative affect implies a collective behavior of the YidC2 cytoplasmic domains in the context of the YidC1 backbone that cannot be attributed to a single domain substitution, or pairs of substitutions. Growth of the YidC1 chimeric strains on pH 5.0 agar plates was consistent with that in liquid media (Supplementary Figure 7), although differences among strains were not as pronounced, possibly because plates were grown in 5% v/v CO_2_ saturated air rather than anaerobically.

The impact of domain swapping on acid tolerance was even more apparent when YidC1 cytoplasmic domains were introduced into YidC2 (Figure 4D). With the exceptions of the simultaneous exchange of the YidC1 C1 loop and tail into YidC2 (**YidC2**-C1,T) or the YidC1 C2 loop and tail into YidC2 (**YidC2**-C2,T), all other combinations of domain substitutions resulted in worsened growth of the YidC2 chimeric strains. The negative impact of the YidC1 C1 loop was ameliorated, and growth of the chimeric YidC2 strain was notably enhanced, when the YidC1 tail was exchanged as well (**YidC2**-C1,T). Simultaneous substitution of YidC1’s C2 loop and tail with those of YidC2 (**YidC2**-C2,T) also alleviated the negative impact of single substitution of the C2 loop (**YidC2**-C2). However, simultaneous transfer of all three of YidC1 cytoplasmic domains into YidC2 (**YidC2**-C1,C2,T) again resulted in a seriously impaired chimeric insertase unable to contend with acid stress. Taken together, these results indicate specific functional attributes of YidC2 compared to YidC1 cytoplasmic domains that must be expressed in the proper combination and context to achieve optimal functional activity.

Exchange of any combination of YidC2 cytoplasmic domains into YidC1 had little to no effect on the ability of the YidC1 chimeric strains to grow in the presence of high salt in liquid culture (Figure 4E), or on agar plates (Supplementary Figure 7). In stark contrast, introducing YidC1 cytoplasmic domains into YidC2 had substantial negative effects on the ability of the YidC2 chimeric strains to grow during osmotic stress (Figure 4F and Supplementary Figure 7), the only exception being co-substitution of the YidC1 C1 loop and tail-domains into YidC2 (**YidC2**-C1,T) which modestly enhanced growth. The most detrimental impact on growth in high salt was observed when only the C2 loop of YidC1 was introduced into YidC2 (**YidC2**-C2). This effect was partially alleviated when the C2 loop was exchanged in combination with the YidC2 C1 loop or C-terminal tail (**YidC2**-C1,C2 or **YidC2**-C2,T). The chimeric strain harboring YidC2 with all three cytoplasmic domains exchanged for those of YidC1 (**YidC2**-C1,C2,T) was only minimally impacted during growth in the presence of high salt when compared to ectopic expression of unmodified *yidC2*. Thus, the results of domain swapping experiments vary considerably depending on the phenotype being evaluated, as well as on the backbone of the insertase under study. This information is expected to become even more enlightening as YidC1- compared to YidC2-specfic substrates responsible for predominant Δ*yidC1* and Δ*yidC2* mutant phenotypes are identified in future studies. Collectively, the results shown in Figure 4 suggest optimal function when the C1 loop and C-terminal tail are paired from the same paralog; however, that beneficial pairing can be obfuscated when all three domains are co-transferred.

### YidC1 Is Specifically Required During *Streptococcus mutans* Growth in Excess Zinc

**FIGURE 5:**
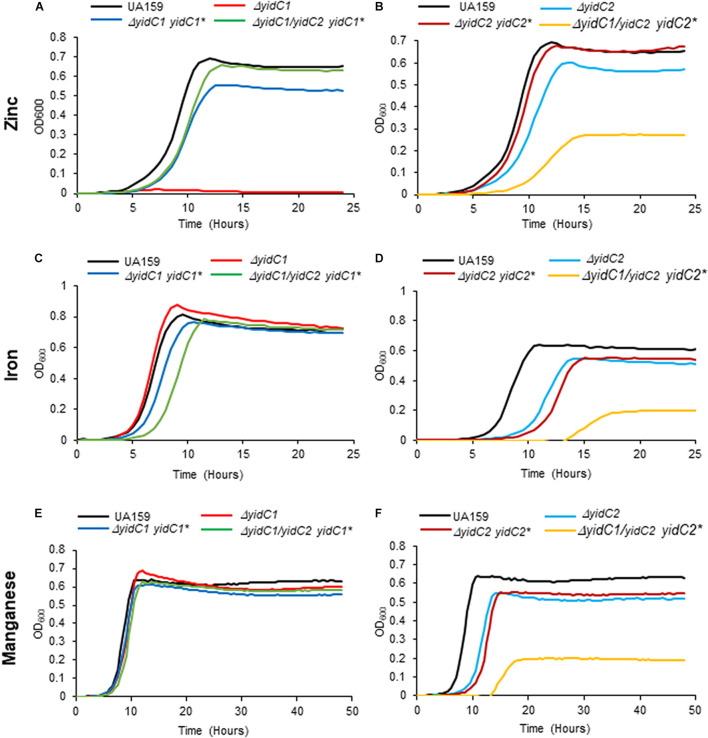
Evaluation of growth of *S. mutans* wild-type, Δ*yidC1*/*2* mutants, and ectopic expression strains under excess transition metal conditions. Growth of the wild-type UA159 parent strain compared to Δ*yidC1* **(A,C,E)** or Δ*yidC2* **(B,D,F)** deletion mutants, and strains complemented by ectopic expression (*) from the *gtfA* locus of *yidC1* or *yidC2* in single Δ*yidC1/2* or double Δ*yidC1/DyidC2* backgrounds. **(A,B)** Growth in THYE, pH 7, supplemented with 2.5 mM ZnCl_2_ under aerating conditions. **(C,D)** Growth in THYE, pH 7, supplemented with 5 mM MnCl_2_ under aerating conditions. **(E,F)** Growth in THYE, pH 7, supplemented with 5 mM MnCl_2_ under aerating conditions. Growth measurements were carried out at least three times with each sample assayed in triplicate. Data shown are mean replicates of an independent experiment.

Our recent membrane proteomic analysis of Δ*yidC1* and Δ*yidC2* deletion mutants, as well as characterization of YidC1 and YidC2 interactomes, suggested a potential involvement of the YidC insertases in metal homeostasis (Supplementary Table 5; Mishra et al., 2019; Lara Vasquez et al., 2021). This is a logical supposition given the membrane localization of integral components of metal ion import and efflux systems. We therefore evaluated growth and survival of Δ*yidC1* and Δ*yidC2* mutants, and ectopic expression strains, in media supplemented with salts of transition metals including zinc, iron, and manganese (Figure 5 and Supplementary Table 4). These growth experiments were carried out under aerated conditions since transition metals are redox-active and their toxicity is therefore linked to oxygen (Storz and Imlay, 1999; Moore and Helmann, 2005). The most striking result of these experiments was a complete inability of the Δ*yidC1* deletion strain to grow in the presence of excess Zn(II) (Figure 5A). Zn(II) is a highly abundant and tightly regulated transition metal that acts as a cofactor for multiple bacterial proteins [reviewed in Chandrangsu et al. (2017)]. Results were similar on agar plates containing 2.5 mM ZnCl_2_ (Supplementary Figure 8). Ectopic expression of *yidC1* in the Δ*yidC1* and Δ*yidC1*/Δ*yidC2* backgrounds was largely able to complement the Zn toxicity phenotype. In contrast, deletion of *yidC2* had far less impact on the ability of *S. mutans* to grow in the presence of excess Zn(II) (Figure 5B). Deletion of *yidC1* had no obvious impact on *S. mutans* growth under conditions of Fe(II) or Mn(II) excess (Figures 5C,E), while growth of the Δ*yidC2* was modestly diminished under these conditions (Figures 5D,F). Growth of the Δ*yidC1*/Δ*yidC2-yidC1* ectopic expression strain was somewhat diminished compared to the UA159 parent strain in the presence of excess Fe(II). In contrast, the strain engineered to express ectopic *yidC2*, while viable in the presence of excess Zn(II), Fe(II), or Mn(II), was severely growth-impaired under these conditions (Figures 5B,D,F). The ability of this strain, which lacks endogenous or ectopic *yidC1*, to grow at all in the presence of excess Zn(II) was initially surprising, but can be explained if the YidC1 and YidC2 paralogs have differential effects on import of particular metals as opposed to efflux. For example, if YidC1 is responsible for insertion of a critical zinc exporter, then inefficient insertion of a metal importer by ectopic expression of *yidC2* would render the cells less susceptible to normally toxic levels of Zn(II) in the extracellular environment. While identification of specific membrane-localized importer vs. exporter substrates is beyond the scope of the current study, evaluation of metal toxicity provides another useful phenotypic property to assess the impact of cytoplasmic domain swaps on YidC1 and YidC2 behavior compared to the unmodified insertases.

**FIGURE 6:**
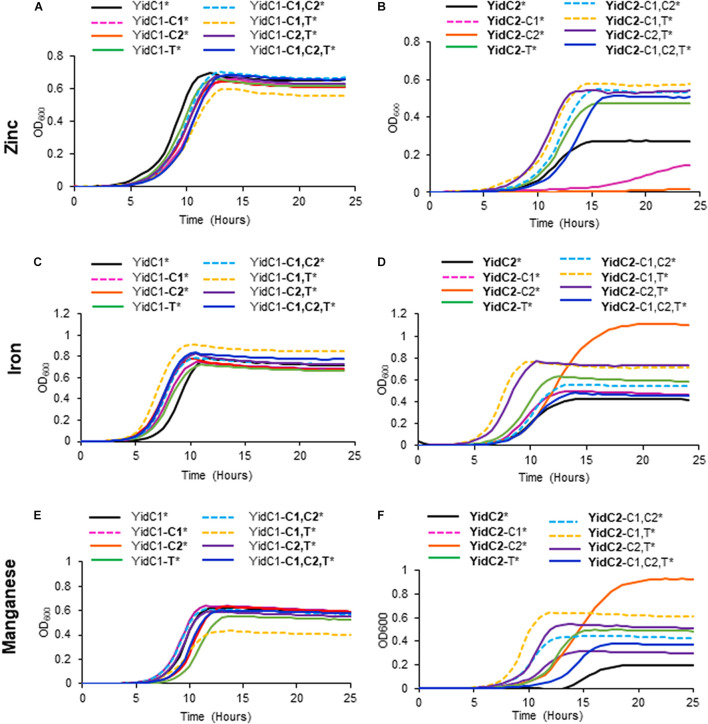
Effect of YidC1/2 cytoplasmic domain substitutions on *S. mutans* growth under excess transition metal conditions. Growth of strains complemented by ectopic expression (*) from the *gtfA* locus of unmodified *yidC1* **(A,C,E)** or *yidC2* **(B,D,F)** compared to corresponding cytoplasmic domain (CD) swap variants in a double Δ*yidC1*/Δ*yidC2* background. **(A,B)** Growth in THYE, pH 7, supplemented with 2.5 mM ZnCl_2_ under aerating conditions. **(C,D)** Growth in THYE, pH 7, supplemented with 5 mM MnCl_2_ under aerating conditions. **(E,F)** Growth in THYE, pH 7, supplemented with 5 mM MnCl_2_ under aerating conditions. Growth measurements were carried out at least three times with each sample assayed in triplicate. Data shown are mean replicates of an independent experiment. Regular text indicates YidC1 backbone or CD. Bold text indicates YidC2 backbone or CD.

Substitution of the cytoplasmic domains of YidC2 into YidC1 had little impact on growth of the chimeric strains in Fe(II), Mn(II), or Zn(II) compared to the unmodified YidC1 control, except that the YidC1-**C1,T** variant grew slightly less well in the presence of excess Zn(II) and slightly better in the presence of excess Fe(II) than the other strains (Figures 6A,E). This strain also achieved a notably lower cell yield during growth in excess Mn(II), and is the first example thus far whereby a homologous pairing of C1 and C-terminal tail domains was apparently detrimental. Overall, these results demonstrate that YidC1 supports the growth of *S. mutans* in the presence of high levels of the metals tested, and that this paralog’s function in this regard is not severely impacted by exchange of its cytoplasmic domains with those of YidC2. The lower cell yield of the YidC1-**C1,T** variant during growth in Mn(II) could stem from increased import or decreased export, or both, of this particular metal. In the case of strains harboring chimeric YidC2 variants, all combinations of single, double, and triple domain swaps of YidC1 cytoplasmic domains into YidC2 improved growth in the presence of Mn(II), with the chimeric **YidC2**-C2 strain achieving a notably higher cell yield (Figure 6F). Results were similar in the presence of excess Fe(II) (Figure 6D), suggesting that overlapping import/efflux systems may contribute to Mn(II) and Fe(II) homeostasis, and that YidC1 cytoplasmic domains, particularly the C2 loop, are more effective than those of YidC2 in the insertion of relevant substrates that limit toxicity by these two metals. The impact of the chimeric YidC2 variants in the presence of excess Zn(II) was strikingly different than in the presence of excess Fe(II) or Mn(II) (Figure 6B). This suggests that *S. mutans* contends with Zn(II) in a different way than it does the other two metals. In this case, incorporation of either the YidC1 C1 loop (**YidC2**-C1), or C2 loop (**YidC2**-C2), into YidC2 severely diminished the ability of the variant insertases to contend with toxic levels of Zn(II). In contrast, all other combinations of domain exchanges substantially improved growth in excess Zn(II). Thus, when the YidC1 C1 and C2 loops were exchanged together, or in combination with the YidC1 C-terminal tail, the net effect on the YidC2 chimeric polypeptides was to enable the cells to better contend with a high level of environmental Zn(II)- again either due to diminished uptake or increased export of this particular metal. The amelioration of the detrimental impact of single substitution of the YidC1 C1 or C2 into YidC2 by co-exchange with other potentially interactive cytoplasmic domains highlights the likely importance of cooperative effects of the cytoplasmic domains of each insertase to accomplish appropriate membrane protein insertion to support specific cellular activities.

### YidC1 and YidC2 Possess Distinct Interdomain Salt Bridges Whose Disruption Diminishes YidC1- and YidC2-Associated Activities

**FIGURE 7:**
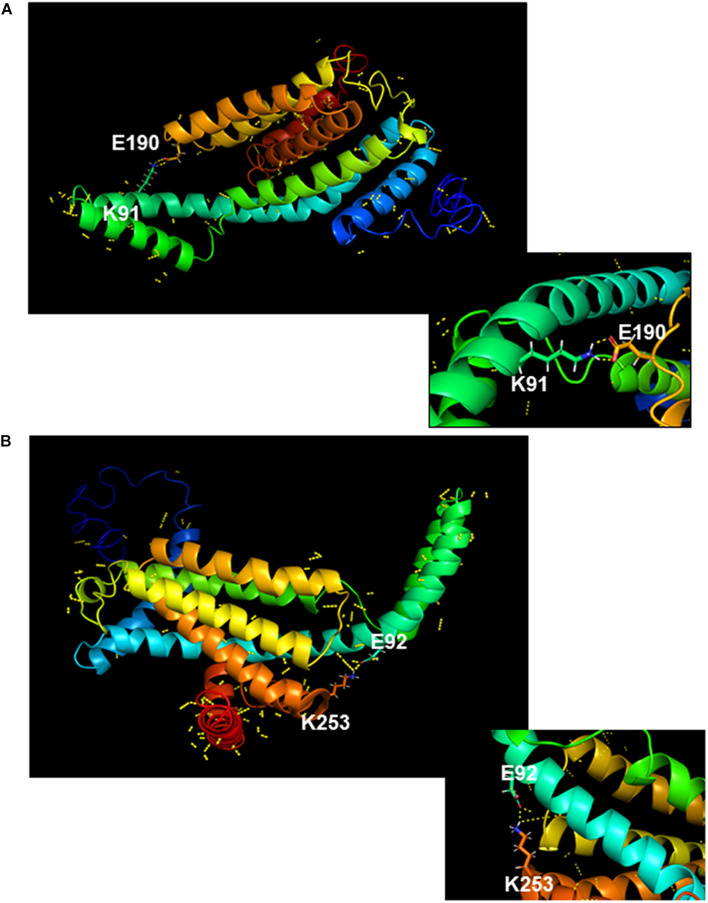
*In silico* modeling of salt bridge formation between cytoplasmic domains of YidC1 or YidC2. **(A)** Putative polar contacts on the side chains of YidC1 (yellow) were mapped and displayed using the Pymol Plugin. The salt bridge between residues K91 and E190 is shown enlarged in the box. **(B)** Putative polar contacts on the side chains of YidC2 (yellow) were mapped and displayed using the Pymol Plugin. The salt bridge between residues E92 and K253 is shown enlarged in the box.

As detailed above, phenotypic characterization of strains producing chimeric YidC1 and YidC2 variants demonstrated preferences for combinatorial replacement of certain cytoplasmic domains over individual substitutions suggesting specific functional inter-domain interactions. Salt bridges (electrostatic interaction between charged residues) often play an important role in the conformational stability of proteins (Ban et al., 2019). All three cytoplasmic domains of YidC1, as well as those of YidC2, contain multiple charged residues that could potentially be involved in formation of salt bridges. We used an *in silico* approach to identify those amino acid residues within the YidC1 and YidC2 cytoplasmic domains most likely to form salt bridges, and measured the distance between the putative donor and acceptor atoms of the residues involved (Supplementary Table 6). Potential intra-domain interactions were identified within the C1 loop and C-terminal tail of YidC1, and within the C1 loop, C2 loop, and C-terminal tail of YidC2. In addition, putative inter-domain interactions between YidC1 amino acid residues K91 (C1 loop) and E190 (C2 loop) (Figure 7A), and YidC2 residues E92 (C1 loop) and K253 (C-terminal tail) (Figure 7B), were identified. The predicted distances between the side chains of both of these pairs of charged residues were <3Å, supporting the likelihood of their interaction. Multiple sequence alignment of YidC1s from various oral streptococci showed that both the K91 and E190 residues are highly conserved (Supplementary Figure 2). Alignment of YidC2 sequences from various oral streptococci showed YidC2 E92 and K253 also to be highly conserved (Supplementary Figure 2). Therefore, we hypothesized that these residues in particular are decisive contributors to the respective functions of YidC1 and YidC2.

**FIGURE 8:**
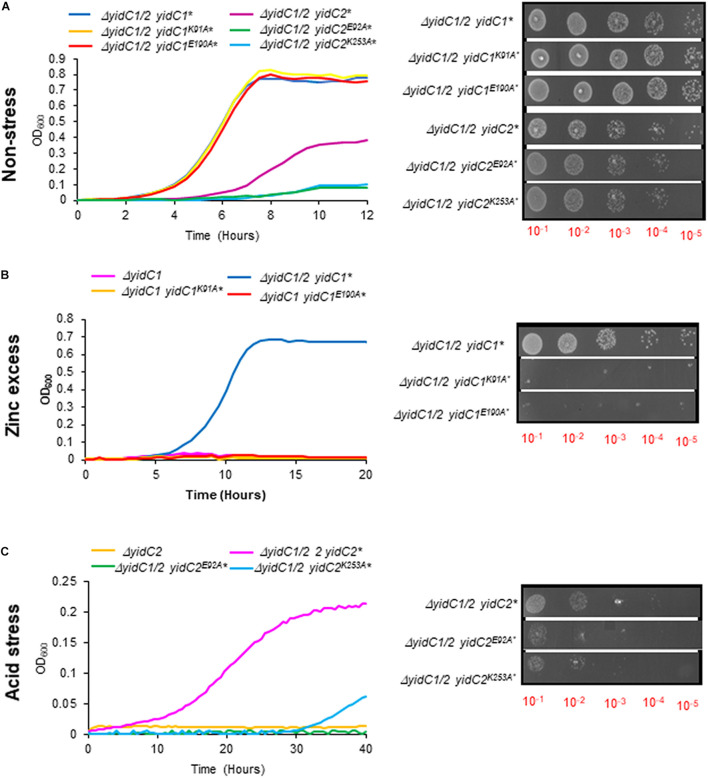
Growth kinetics and efficiency of plating assay of *S. mutans* strains expressing *yidC1* or *yidC2* containing point mutations of residues predicted to contribute to salt bridge formation. Growth of strains complemented by ectopic expression (*) from the *gtfA* locus of unmodified *yidC1, yidC2*, or *yidC1* or *yidC2* containing the indicated point mutations, in a double Δ*yidC1*/Δ*yidC2* background under **(A)** non-stress (THYE, pH 7.0), **(B)** zinc excess (THYE containing 2.5 mM ZnCl_2_), or **(C)** acid stress (THYE, pH 5.0) conditions.

To examine the functional relevance of key residues predicted by *in silico* analysis to form stabilizing interactions within the YidC1 or YidC2 structures, we used site-directed mutagenesis to change each of these charged residues to an alanine. The modified *yidC1* or *yidC2* genes were expressed similarly to the chimeric genes under control of the *gtfA* promoter in a Δ*yidC1*/Δ*yidC2* background for phenotypic characterization. Measurement of growth kinetics under non-stress conditions showed that the strains engineered to produce YidC2E92A or YidC2K253A were clearly affected compared to the unmodified YidC2 control strain. The negative impact of the YidC2 E92 and K253 mutations on growth of the *yidC2* ectopic expression strain was also observed by dilution plating of log-phase cultures. In contrast, strains producing YidC1K91A or YidC1E190A did not show any obvious difference in growth compared to ectopic expression of unmodified *yidC1* (Figure 8A). Thus, the YidC1 point mutations did not appear to impact its general housekeeping functions. However, when the ability of the point mutant strains to tolerate environmental stress conditions reflective of previously identified YidC1- and YidC2-associated functions was assessed, the apparent importance of the putative salt bridges was striking. The ability of ectopically expressed *yidC1* to support *S. mutans* growth in the presence of excess zinc was clearly abolished by the K91A and E190A point mutations (Figure 8B). Next, we measured the impact of point mutations within YidC2 on growth of *S. mutans* in THYE acidified to pH 5.0 (Figure 8C). Both the E92A and K253A point mutations destroyed the ability of YidC2 to contend with acid stress. Taken together, these results confirm that the residues predicted to form salt bridges within YidC1 and YidC2, respectively, do in fact contribute to the respective functional activities of each paralog under specific environmental conditions.

## Discussion

YidC belongs to the Oxa1/Alb3/YidC family of proteins present in all three domains of the life, and plays a pivotal role in the proper folding and insertion of membrane proteins in bacteria (Dalbey and Kuhn, 2015; Chen et al., 2019). When discovered in *E. coli*, YidC was demonstrated to be essential for viability (Samuelson et al., 2000). Later, discovery of two different paralogs in Gram-positive bacteria, including *B. subtilis* and *S. mutans*, showed that at least one YidC is required for growth and survival under various environmental conditions, although they are not equally effective (Errington et al., 1992; Tjalsma et al., 2003; Hasona et al., 2005; Saller et al., 2011; Palmer et al., 2012; Mishra et al., 2019; Zhao et al., 2019). Subsequent proteomic analysis of *S. mutans* membrane preparations from mutants lacking either *yidC1* or *yidC2*, coupled with characterization of the YidC1 compared to YidC2 interactomes, further revealed paralog-specific activities as well as overlap in their functional capabilities (Mishra et al., 2019; Lara Vasquez et al., 2021). Thus, when one paralog is eliminated, the residual paralog must accomplish many of the basic housekeeping needs of the cell. However, each paralog cannot fully accomplish the specific functions mediated by the other, thereby manifesting in characteristic phenotypic consequences. As expected for paralogous bacterial proteins, divergent SmYidC1 and SmYidC2 amino acid sequences have retained low sequence identity (∼30%). Nevertheless, their similar membrane topologies and largely superimposable predicted 3D structures are consistent with their high degree of functional overlap. Other than disparate roles for the C-terminal tails (Palmer et al., 2012), specific structural features responsible for distinct YidC1- and YidC2-associated functional attributes have not been characterized. The absence of SmYidC1 and SmYidC2 crystallographic data, the numerous yet unidentified paralog-specific membrane-localized substrates, and the current lack of experimental methods to carry out membrane protein insertion assays using exclusively components of Gram-positive translation and translocation machineries have hindered our understanding of YidC1- vs. YidC2-specific structure-function features. In this study, we utilized insights obtained from *in silico* sequence-structure analyses to engineer all possible cytoplasmic domain-swapped versions of YidC1/2 and tested growth of the chimeric variants under those environmental conditions known (acid, high salt), or predicted (excess metal), to contribute to paralog-specific phenotypes.

### Cytoplasmic Domains Impart Distinct Functional Attributes to SmYidC1 and SmYidC2

An important breakthrough in the field of YidC structure-function occurred when crystal structures of BhYidC2 and *E. coli* YidC (EcYidC) were determined in 2014 (Kumazaki et al., 2014b,c). Both solved structures demonstrated the presence of a hydrophilic groove formed by the five core TMDs, which was proposed as a conserved structural feature of all YidCs. Structuralanalyses of both species’ YidCs further suggested their C1 loops as a primary point of contact for incoming substrates (Kumazaki et al., 2014a). Another unrelated study utilized EcYidC mutants containing segmental deletions of the C1 loop and demonstrated the significance of the second-half of this domain in rescuing a YidC-depletion strain; although this work proposed that the precise sequence of C1 is not essential for EcYidC function (Chen et al., 2014). More recently, Koch and coworkers used formaldehyde cross-linking of *E. coli* cells to demonstrate that the YidC C1 loop interacts with the signal recognition particle (SRP), the SRP receptor FtsY, SecY, SecE, and SecG, with a stronger interaction identified with SRP compared to SecYEG (Petriman et al., 2018). Unlike its C1 loop, the BhYidC2 C2 loop was not structurally resolved by X-ray crystallography (Kumazaki et al., 2014c). Molecular dynamic (MD) simulations, however, predicted a contribution of the C2 loop in stabilizing protein structure, especially in reducing flexibility of the C1 loop by formation of an inter-domain salt bridge between D205 (C2) and K109 (C1) (Harkey et al., 2019). Structural analysis of BhYidC2 also revealed that its C1 loop is assessable to the cytoplasm, while the smaller C2 loop appears to be embedded within the lipid bilayer and probably involved in an interaction with lipid heads.

Since the discovery of dual YidC paralogs in the overwhelming majority of Gram-positive bacteria, it has been of broad interest to understand how they are structurally and functionally distinguished. Distinctive lengths and charge distribution of their cytoplasmic tails is a striking feature. Previous work demonstrated the significance of the SmYidC2 tail with respect to its contribution to acid tolerance; however, the underlying mechanism for this result was not fully understood (Palmer et al., 2012). Driessen and coworkers used purified SmYidC1 and SmYidC2 to demonstrate that both of their tails appear to be involved in interactions with ribosomal proteins; although this work was carried out using *E. coli* ribosomes (Wu et al., 2013). Of note, the longer tails of marine Gram-negative bacteria such as *Rhodopirellula baltica* and *Oceanicaulis alexandrii* interacted with *E. coli* ribosomal proteins, whereas *E. coli* YidC with a shorter C-terminal tail failed to do so (Seitl et al., 2014). We analyzed the respective interactomes of YidC1 and YidC2, including assessment of the binding partners of their C-terminal tails by 2D-difference gel electrophoresis (DIGE) (Lara Vasquez et al., 2021). These data clearly suggested that the isolated C-terminal tails can interact with multiple ribosomal proteins but do not appear to play an important role in recognizing and binding substrates. Characterization of the YidC1/2 protein interactomes showed that of YidC1 to be larger than that of YidC2, and further identified YidC1-SecYEG and YidC2-SRP interactions in *S. mutans* (Lara Vasquez et al., 2021). Bacterial two-hybrid experiments revealed that YidC1 could interact with both SecY and Ffh, while YidC2 could only interact with the SRP protein Ffh (Lara Vasquez et al., 2021). This explained in part, why individual deletion of *yidC2* or *ffh* results in similar growth defects and stress-sensitivity profiles, and co-deletion of *yidC2* and *ffh* is lethal (Hasona et al., 2005; Mishra et al., 2019). Taken together with prior interactome data, and the *in silico* sequence-structure analyses performed in the current study, we hypothesized that their cytoplasmic domains play pivotal roles in conferring functional differences between *S. mutans* YidC1 and YidC2. The data we obtained support this hypothesis.

### YidC1 Structure Is More Functionally Resilient That of YidC2

*In silico* analyses showed higher RMSD values, and therefore greater structural deviations from unmodified YidC1 for most of the chimeric YidC1 variants. Yet strains harboring chimeric YidC1 did not manifest significant growth phenotypes under numerous experimental conditions. This suggests that YidC1 can accommodate multiple structural perturbations without loss of function, or in other words, that its structural malleability is associated with functional plasticity. In contrast, *in silico* analyses of chimeric YidC2 variants showed less structural deviation from unmodified YidC2, with most domain substitutions resulting in impaired YidC2 function as manifested by an inability to support bacterial growth under the conditions tested. This suggests that the more rigid nature of YidC2 compared to YidC1 constrains its ability to mediate insertion of the membrane proteins necessary for bacterial growth and tolerance to environmental stress when its cytoplasmic domains are exchanged for those of YidC1.

### YidC1 and YidC2 Cytoplasmic Domains Possess Distinct Intra- and Inter-Domain Interactions

An interesting outcome of the current work was the preference for certain combinations of cytoplasmic domains over others, which was especially notable among the chimeric YidC2 variants. The results of *in silico* analyses of chimeras compared with unmodified YidC1 and YidC2, as evidenced by small (<3 Å) RMSD values, further suggests that certain combinatorial substitutions result in a propensity for the tertiary structure of a given chimera to more closely align with YidC1 or YidC2. In light of *in silico* structure predictions, combined with insight gained from phenotypic assays, we hypothesized that specific interactions between cytoplasmic domains were responsible for particular structure-function attributes. In fact, Moradi and coworkers had proposed that formation of a salt-bridge between the C1 and C2 loops of *B. halodurans* YidC2 appears to stabilize the flexible C1 loop (Harkey et al., 2019). When the SmYidC1 and SmYidC2 cytoplasmic domains were evaluated, both paralogs contained multiple oppositely charged residues within close enough proximity (<3 Å) to form salt-bridges (Supplementary Table 6). Most were intra-domain interactions; however, we focused on the two inter-domain interactions identified within YidC1 [K91(C1)-E190(C2)] and YidC2 [E92(C1)-K253(tail)], respectively. Similar to deletion of *yidC2*, the YidC2K253A mutant had a severe growth phenotype. The other three point mutants also recapitulated environmental stress phenotypes associated with *yidC1* or yidC*2* deletion. Hence, the predicted inter-domain salt bridges appear to play important roles in stabilizing YidC1 and YidC2 structures that are necessary for paralog-specific activities.

### Impact of YidC1 and YidC2 on Metal Toxicity

An interesting outcome of the current work was the discovery of a novel *yidC1* elimination phenotype. We previously identified pronounced sensitivity to acid, osmotic, or oxidative stress as a result of *yidC2* elimination (Hasona et al., 2005). In contrast, elimination of *yidC1* had far less obvious consequences apart from a modest impact on maturation of surface proteins and hyperadherence to salivary components (Palmer et al., 2012). However, until now a readily assayable clearcut phenotype associated primarily with *yidC1* elimination had not been observed. Our proteomic characterization of *S. mutans* membrane preparations derived from WT and mutant strains, including Δ*yidC1* and Δ*yidC2*, had suggested overlapping, as well as differential, impacts of elimination of these insertases on the levels of previously characterized, or predicted, membrane-localized metal transporters or regulators (Supplementary Table 5; Mishra et al., 2019). Thus, the survival of *S. mutans ΔyidC1* and Δ*yidC2* mutants, and the behavior of the ectopic-expression chimeric and control strains, in the presence of excess zinc, iron, and manganese were assessed as part of this work. *S. mutans* contains multiple transporters for Fe(II) including FeoABC, the SMU_995-998 system, and the Mn(II) transporters SloABC and MntH (Paik et al., 2003; Galvao et al., 2015; Ganguly et al., 2018). Deletion of *yidC2* had a more apparent impact on *S. mutans* tolerance to Fe(II) and Mn(II) excess than deletion of *yidC1*, with most YidC2-backbone chimeric proteins being negatively impacted by substitution of YidC1 cytoplasmic domains. However, lack of complete complementation of iron and manganese tolerance by ectopic expression of *yidC2* in a Δ*yidC1*/Δ*yidC2* background suggests the existence of regulatory circuits that will require extensive future study.

While deletion of *yidC2* had a slight impact on zinc tolerance, it did not approach that of *yidC1* deletion. Ectopic expression of *yidC1* in a Δ*yidC1*/Δ*yidC2* background almost completely restored growth in excess Zn(II) to wild-type levels. Proteomic data revealed a pronounced decrease in levels of the putative metal transporter Smu_2057 associated with elimination of *yidC1* compared to *yidC2* (Supplementary Table 5). Blast search showed SMU_2057 to be 42% identical and 61% homologous to the *B. subtilis* metal transporting ATPase, PfeT. Mutation of *pfeT* led to improved growth in Zn(II) excess conditions (Gaballa and Helmann, 2002), but the molecular mechanism of the PfeT-dependent increase in growth remains elusive (Guan et al., 2015). It has been proposed to be mediated by mismetallation caused by Zn(II) under aerated conditions when PfeT-dependent iron-efflux is inhibited. The role of SMU_2057 in zinc homeostasis in *S. mutans*, and the potential role of YidC1 in its membrane insertion, will be of much interest in future studies.

### Concluding Remarks

In summary, this work successfully revealed distinct functional contributions of cytoplasmic domains to paralog-specific functions of *S. mutans* YidC1 and YidC2. The novel discovery of functionally significant residues within the *S. mutans* YidC1/2 cytoplasmic domains is important, especially since bacterial YidC TMDs have received more attention in prior structure-function studies given their membrane localization and direct involvement in substrate insertion. This newly acquired knowledge will provide additional insights as YidC1/2-specific substrates and relevant interactions with other components of the protein translocation machinery are identified in *S. mutans* and other Gram-positive bacteria.

## Data Availability Statement

The original contributions presented in the study are included in the article/Supplementary Material, further inquiries can be directed to the corresponding author/s.

## Author Contributions

SM and LJB conceived the experiments, analyzed the data, and wrote the manuscript. SM performed the experiments and collected data. All authors contributed to the article and approved the submitted version.

## Conflict of Interest

The authors declare that the research was conducted in the absence of any commercial or financial relationships that could be construed as a potential conflict of interest.

## Publisher’s Note

All claims expressed in this article are solely those of the authors and do not necessarily represent those of their affiliated organizations, or those of the publisher, the editors and the reviewers. Any product that may be evaluated in this article, or claim that may be made by its manufacturer, is not guaranteed or endorsed by the publisher.

## Footnotes

1
https://weblogo.berkeley.edu/logo.cgi

2
https://web.expasy.org/protparam/
