# Supplementary material for: The Cytoplasmic Domains of Streptococcus mutans Membrane Protein Insertases YidC1 and YidC2 Confer Unique Structural and Functional Attributes to Each Paralog
Source: Front Microbiol. 2021 Nov 2;12:760873. doi: 10.3389/fmicb.2021.760873 (PMC8595059; doi:10.3389/fmicb.2021.760873)
Supplement: Supplementary file 1 [file Presentation_1.PPTX]

## Slide 1
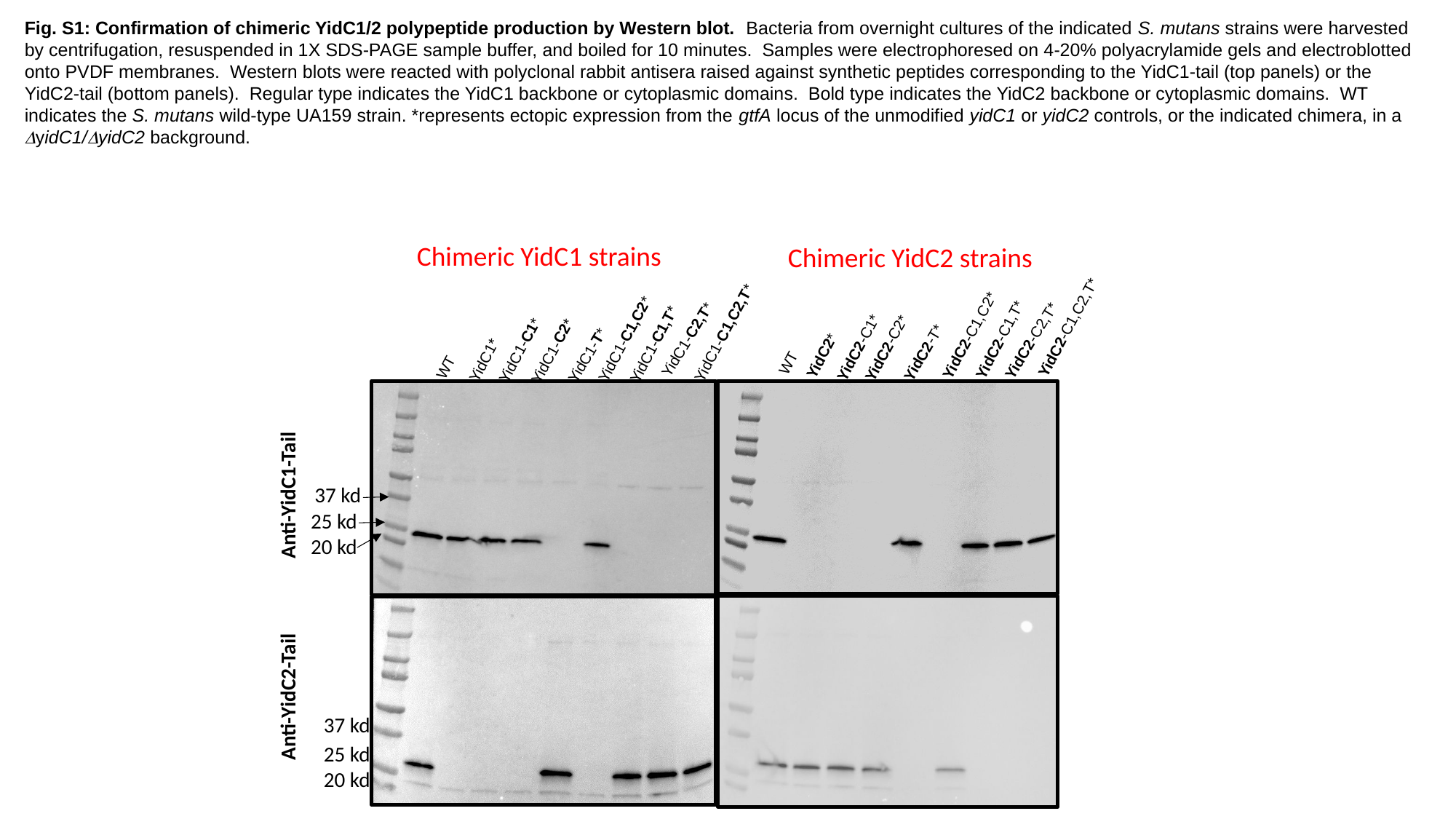

Fig. S1: Confirmation of chimeric YidC1/2 polypeptide production by Western blot. Bacteria from overnight cultures of the indicated S. mutans strains were harvested by centrifugation, resuspended in 1X SDS-PAGE sample buffer, and boiled for 10 minutes. Samples were electrophoresed on 4-20% polyacrylamide gels and electroblotted onto PVDF membranes. Western blots were reacted with polyclonal rabbit antisera raised against synthetic peptides corresponding to the YidC1-tail (top panels) or the YidC2-tail (bottom panels). Regular type indicates the YidC1 backbone or cytoplasmic domains. Bold type indicates the YidC2 backbone or cytoplasmic domains. WT indicates the S. mutans wild-type UA159 strain. *represents ectopic expression from the gtfA locus of the unmodified yidC1 or yidC2 controls, or the indicated chimera, in a DyidC1/DyidC2 background.
Chimeric YidC1 strains
Chimeric YidC2 strains
YidC2-C1,C2*
YidC1-C1,C2*
YidC1-C1,C2,T*
YidC1-C2,T*
YidC2-C1,T*
YidC2-C2,T*
YidC1-C1,T*
YidC2-C1*
YidC2-T*
YidC2-C2*
YidC1-C1*
WT
YidC1-T*
YidC1-C2*
YidC2*
WT
YidC1*
Anti-YidC1-Tail
37 kd
25 kd
20 kd
Anti-YidC2-Tail
37 kd
25 kd
20 kd
YidC2-C1,C2,T*

## Slide 2
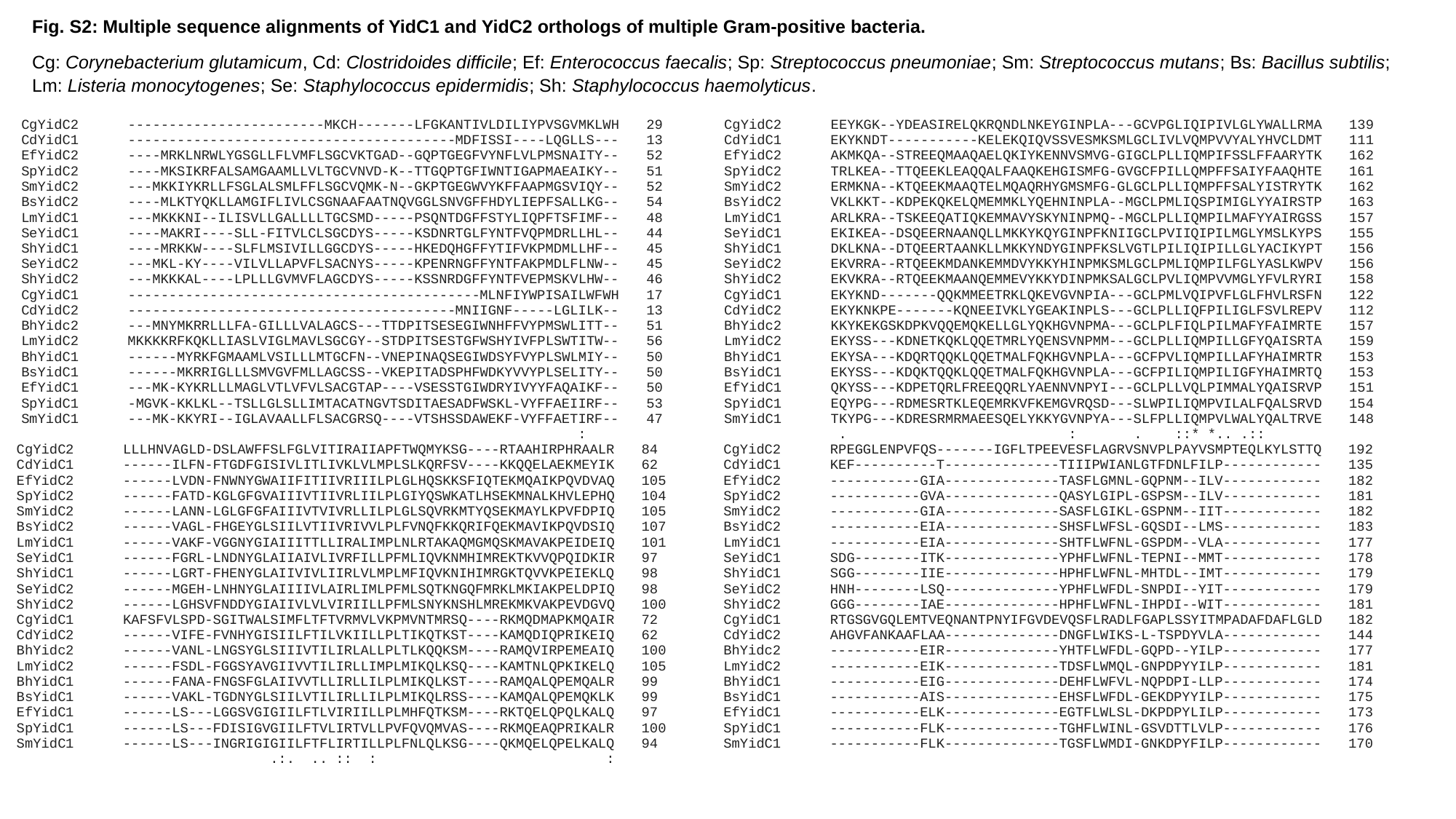

Fig. S2: Multiple sequence alignments of YidC1 and YidC2 orthologs of multiple Gram-positive bacteria.
Cg: Corynebacterium glutamicum, Cd: Clostridoides difficile; Ef: Enterococcus faecalis; Sp: Streptococcus pneumoniae; Sm: Streptococcus mutans; Bs: Bacillus subtilis; Lm: Listeria monocytogenes; Se: Staphylococcus epidermidis; Sh: Staphylococcus haemolyticus.

## Slide 3
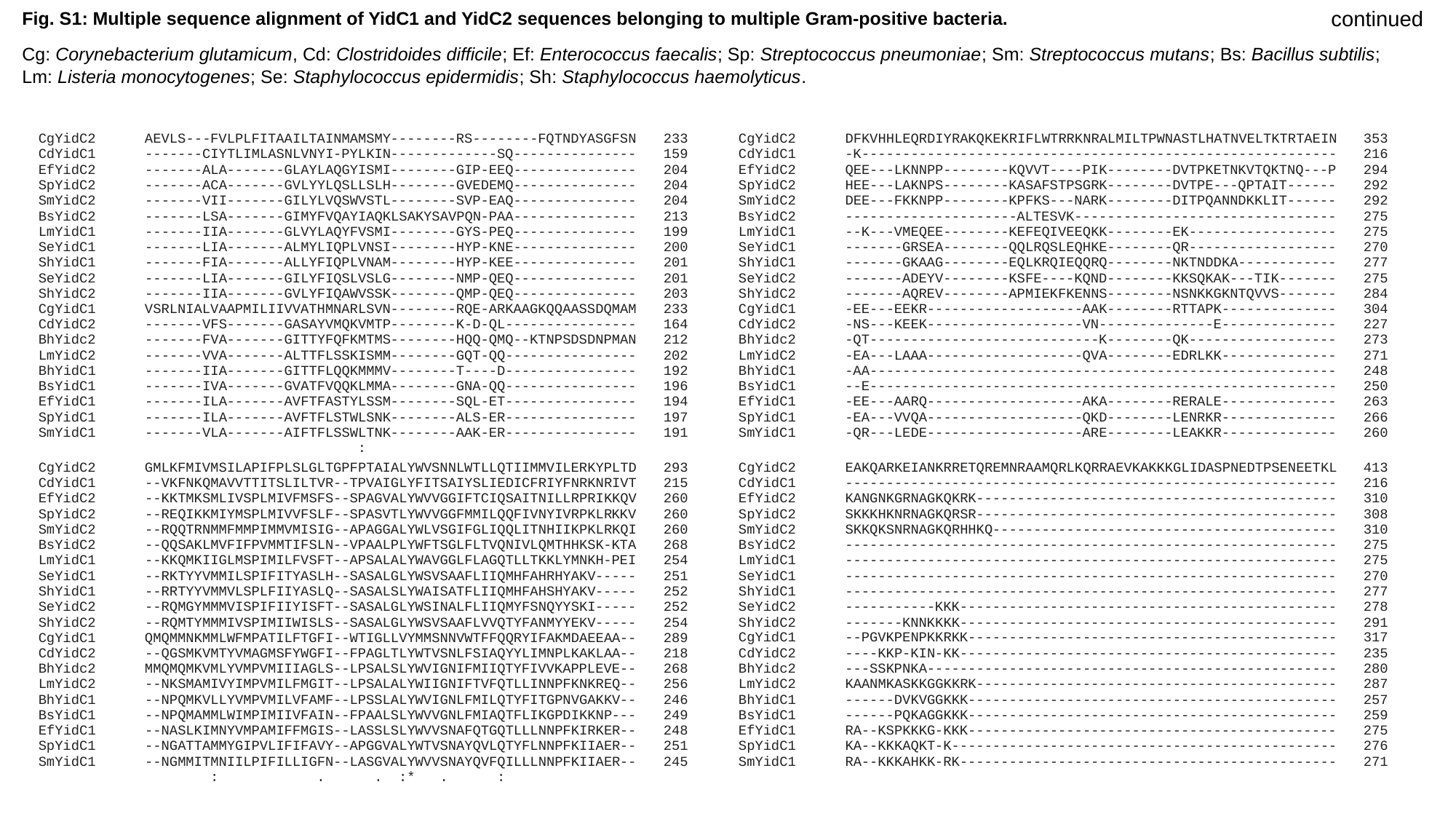

Fig. S1: Multiple sequence alignment of YidC1 and YidC2 sequences belonging to multiple Gram-positive bacteria.
Cg: Corynebacterium glutamicum, Cd: Clostridoides difficile; Ef: Enterococcus faecalis; Sp: Streptococcus pneumoniae; Sm: Streptococcus mutans; Bs: Bacillus subtilis; Lm: Listeria monocytogenes; Se: Staphylococcus epidermidis; Sh: Staphylococcus haemolyticus.
continued

## Slide 4
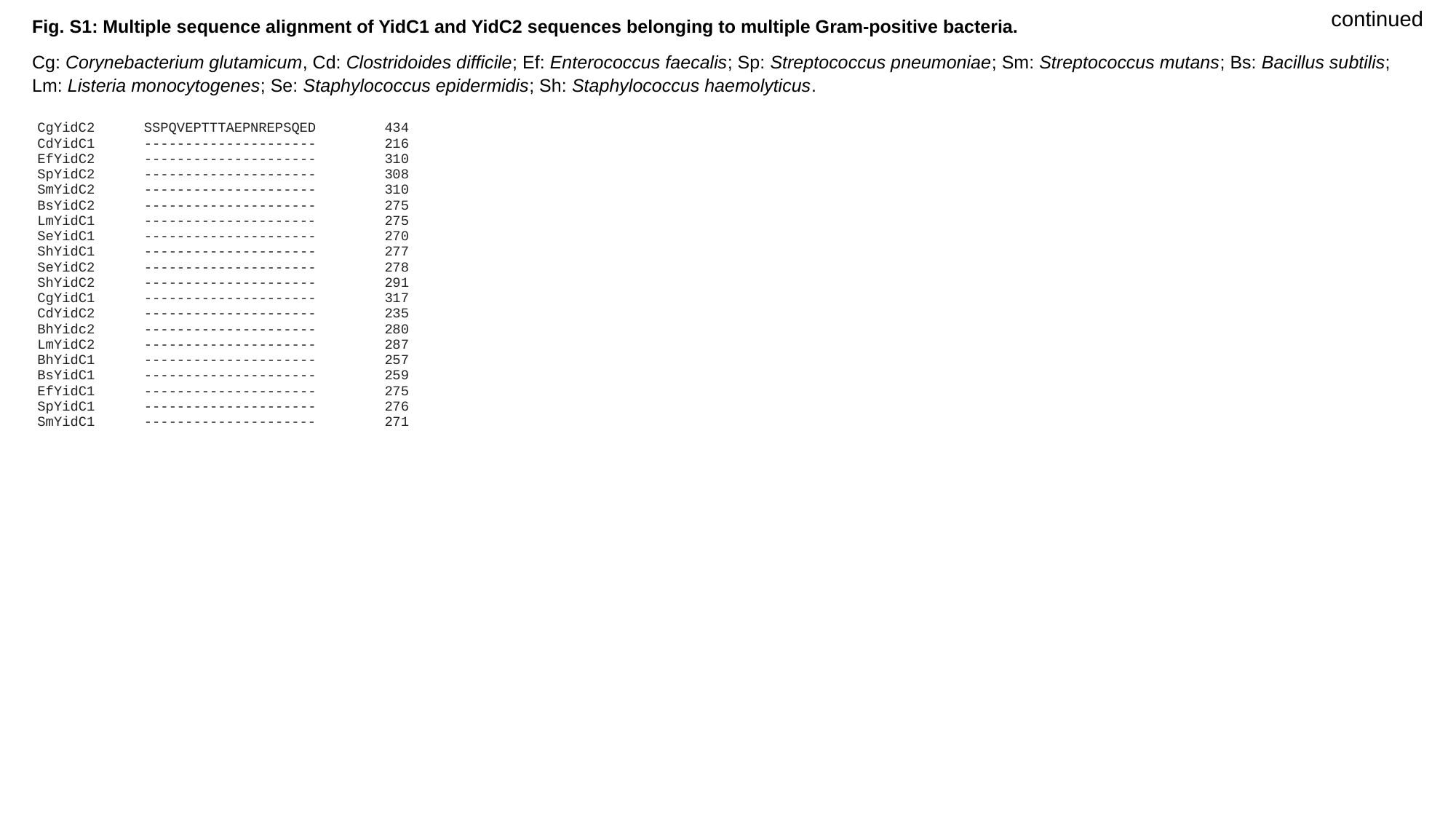

continued
Fig. S1: Multiple sequence alignment of YidC1 and YidC2 sequences belonging to multiple Gram-positive bacteria.
Cg: Corynebacterium glutamicum, Cd: Clostridoides difficile; Ef: Enterococcus faecalis; Sp: Streptococcus pneumoniae; Sm: Streptococcus mutans; Bs: Bacillus subtilis; Lm: Listeria monocytogenes; Se: Staphylococcus epidermidis; Sh: Staphylococcus haemolyticus.

## Slide 5
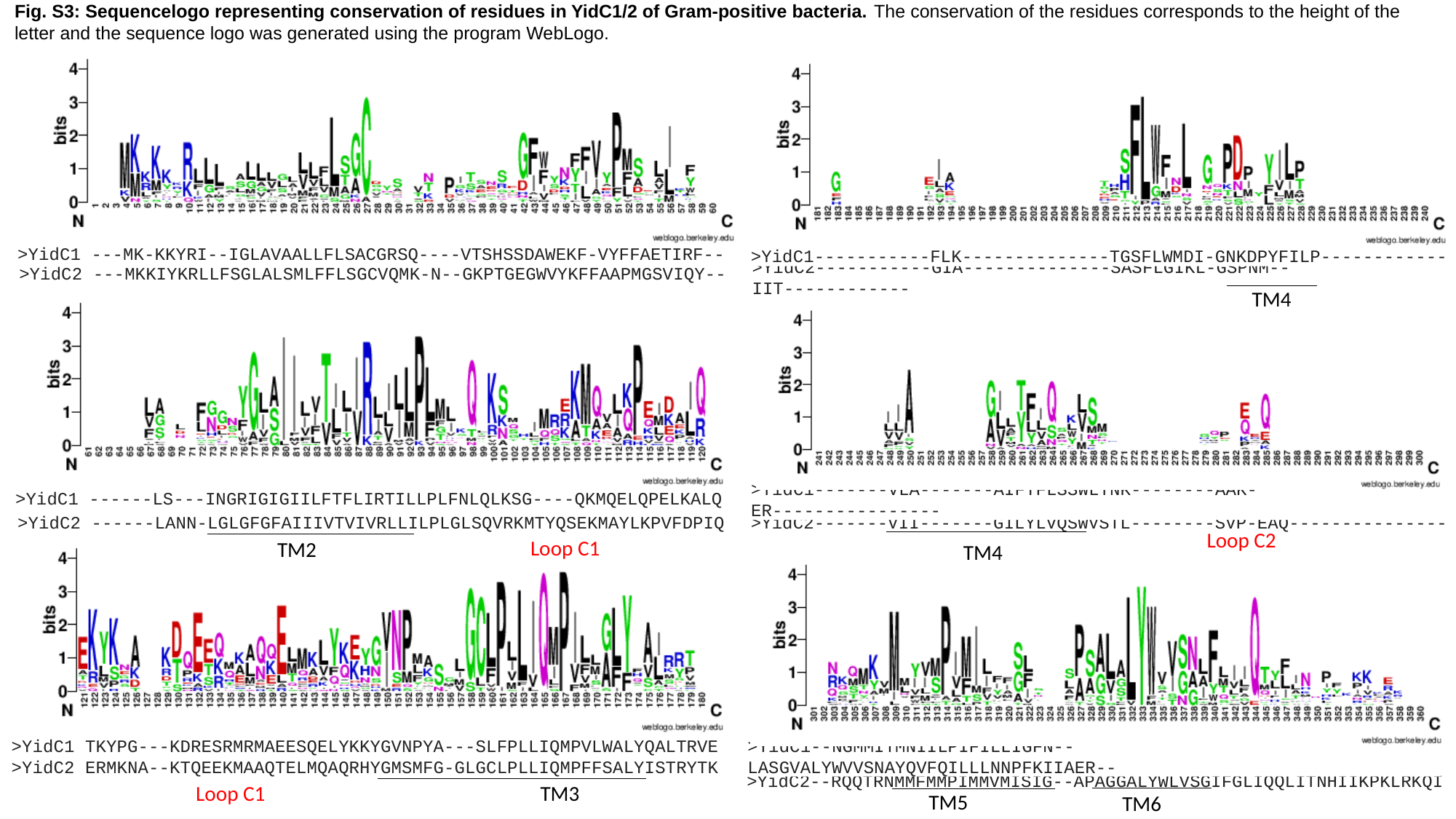

Fig. S3: Sequencelogo representing conservation of residues in YidC1/2 of Gram-positive bacteria. The conservation of the residues corresponds to the height of the letter and the sequence logo was generated using the program WebLogo.
 >YidC1 ---MK-KKYRI--IGLAVAALLFLSACGRSQ----VTSHSSDAWEKF-VYFFAETIRF--
 >YidC2 ---MKKIYKRLLFSGLALSMLFFLSGCVQMK-N--GKPTGEGWVYKFFAAPMGSVIQY--
>YidC1-----------FLK--------------TGSFLWMDI-GNKDPYFILP------------
>YidC2-----------GIA--------------SASFLGIKL-GSPNM--IIT------------
TM4
 >YidC1 ------LS---INGRIGIGIILFTFLIRTILLPLFNLQLKSG----QKMQELQPELKALQ
 >YidC2 ------LANN-LGLGFGFAIIIVTVIVRLLILPLGLSQVRKMTYQSEKMAYLKPVFDPIQ
>YidC1-------VLA-------AIFTFLSSWLTNK--------AAK-ER----------------
>YidC2-------VII-------GILYLVQSWVSTL--------SVP-EAQ---------------
Loop C2
TM2
Loop C1
TM2
TM4
 >YidC1 TKYPG---KDRESRMRMAEESQELYKKYGVNPYA---SLFPLLIQMPVLWALYQALTRVE
>YidC1--NGMMITMNIILPIFILLIGFN--LASGVALYWVVSNAYQVFQILLLNNPFKIIAER--
 >YidC2 ERMKNA--KTQEEKMAAQTELMQAQRHYGMSMFG-GLGCLPLLIQMPFFSALYISTRYTK
>YidC2--RQQTRNMMFMMPIMMVMISIG--APAGGALYWLVSGIFGLIQQLITNHIIKPKLRKQI
Loop C1
TM3
TM5
TM6

## Slide 6
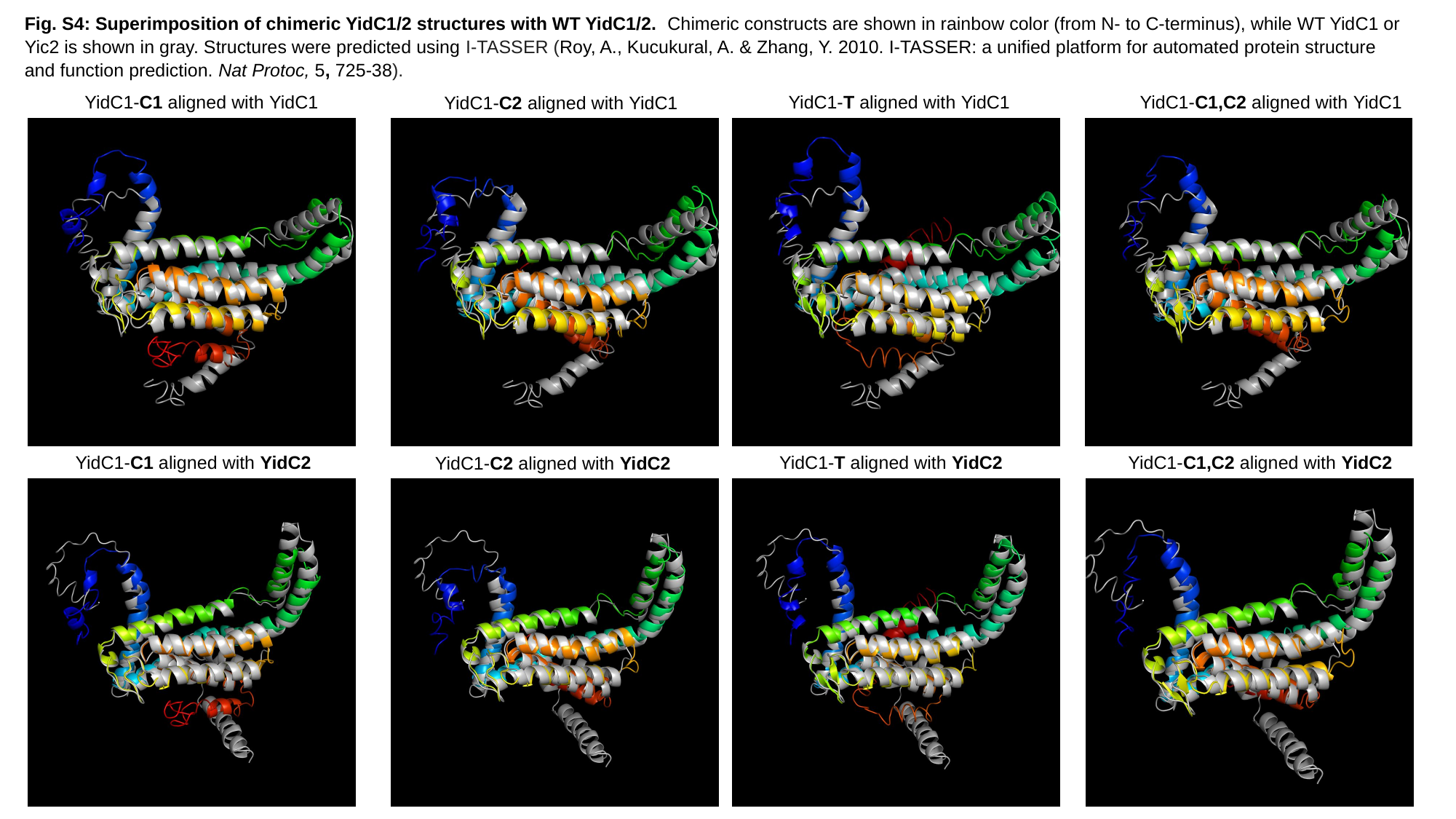

Fig. S4: Superimposition of chimeric YidC1/2 structures with WT YidC1/2. Chimeric constructs are shown in rainbow color (from N- to C-terminus), while WT YidC1 or Yic2 is shown in gray. Structures were predicted using I-TASSER (Roy, A., Kucukural, A. & Zhang, Y. 2010. I-TASSER: a unified platform for automated protein structure and function prediction. Nat Protoc, 5, 725-38).
YidC1-C1 aligned with YidC1
YidC1-T aligned with YidC1
YidC1-C1,C2 aligned with YidC1
YidC1-C2 aligned with YidC1
YidC1-C1 aligned with YidC2
YidC1-T aligned with YidC2
YidC1-C1,C2 aligned with YidC2
YidC1-C2 aligned with YidC2

## Slide 7
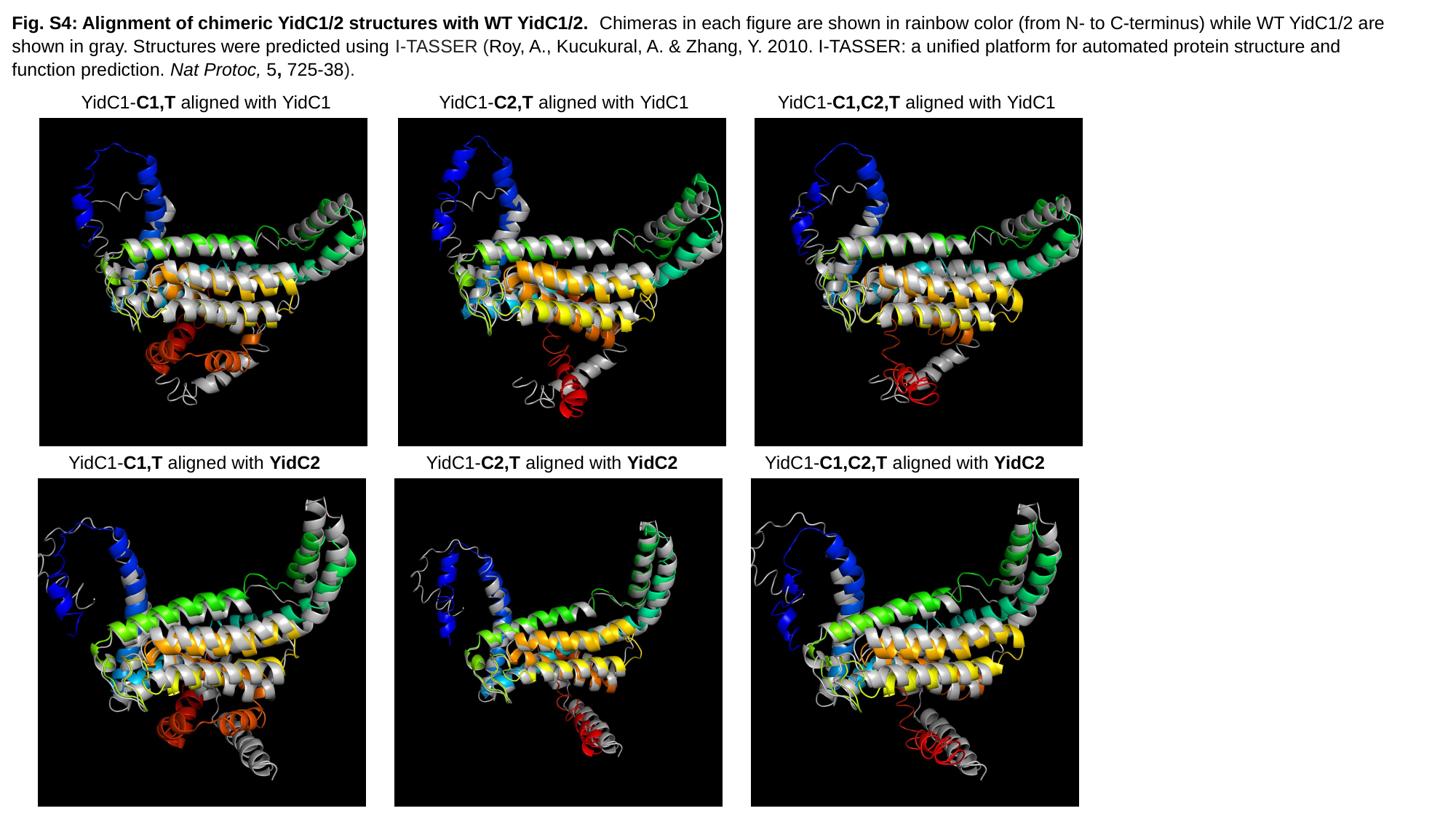

Fig. S4: Alignment of chimeric YidC1/2 structures with WT YidC1/2. Chimeras in each figure are shown in rainbow color (from N- to C-terminus) while WT YidC1/2 are shown in gray. Structures were predicted using I-TASSER (Roy, A., Kucukural, A. & Zhang, Y. 2010. I-TASSER: a unified platform for automated protein structure and function prediction. Nat Protoc, 5, 725-38).
YidC1-C1,T aligned with YidC1
YidC1-C2,T aligned with YidC1
YidC1-C1,C2,T aligned with YidC1
YidC1-C1,T aligned with YidC2
YidC1-C2,T aligned with YidC2
YidC1-C1,C2,T aligned with YidC2

## Slide 8
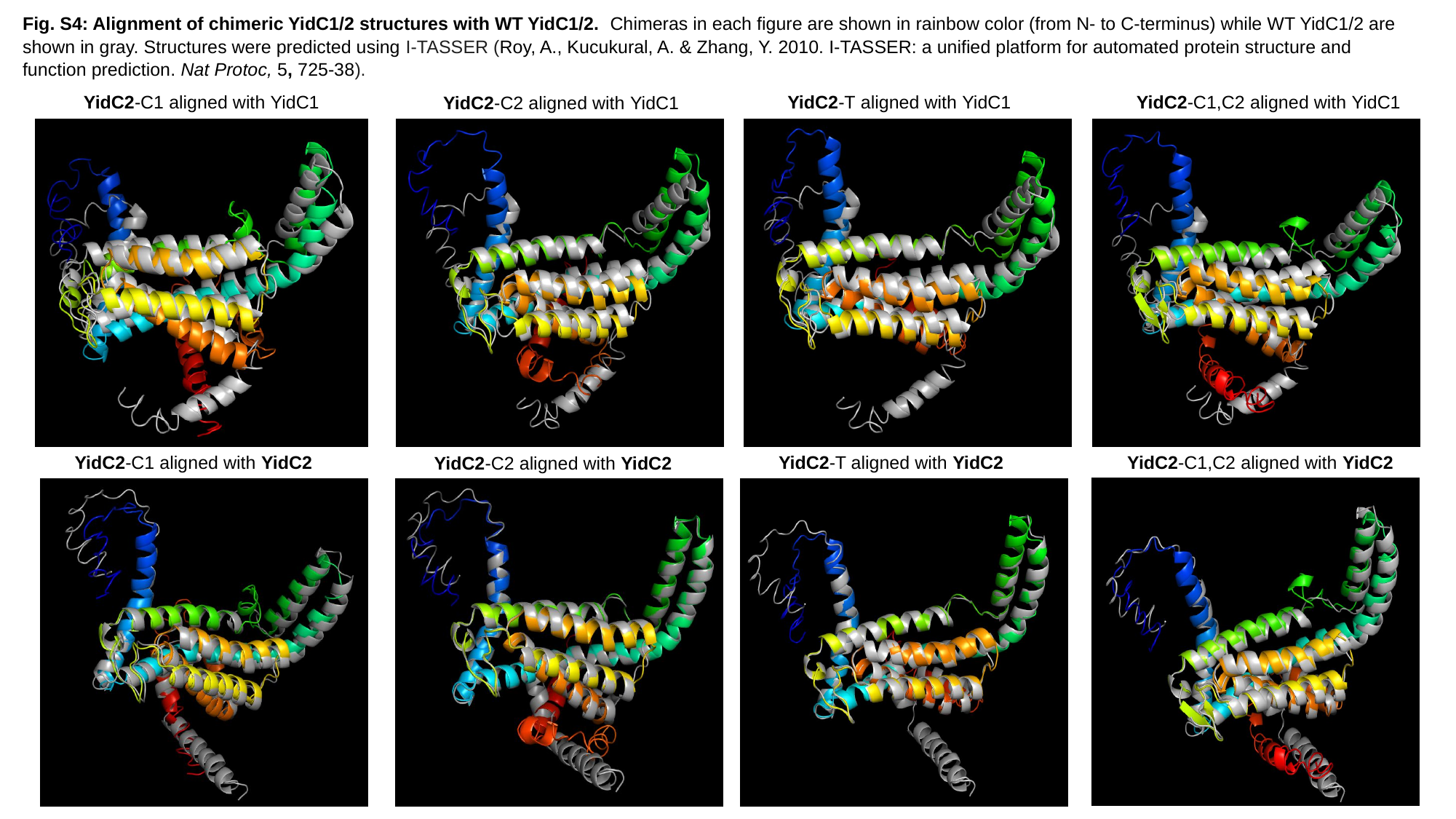

Fig. S4: Alignment of chimeric YidC1/2 structures with WT YidC1/2. Chimeras in each figure are shown in rainbow color (from N- to C-terminus) while WT YidC1/2 are shown in gray. Structures were predicted using I-TASSER (Roy, A., Kucukural, A. & Zhang, Y. 2010. I-TASSER: a unified platform for automated protein structure and function prediction. Nat Protoc, 5, 725-38).
YidC2-C1 aligned with YidC1
YidC2-T aligned with YidC1
YidC2-C1,C2 aligned with YidC1
YidC2-C2 aligned with YidC1
YidC2-C1 aligned with YidC2
YidC2-T aligned with YidC2
YidC2-C1,C2 aligned with YidC2
YidC2-C2 aligned with YidC2

## Slide 9
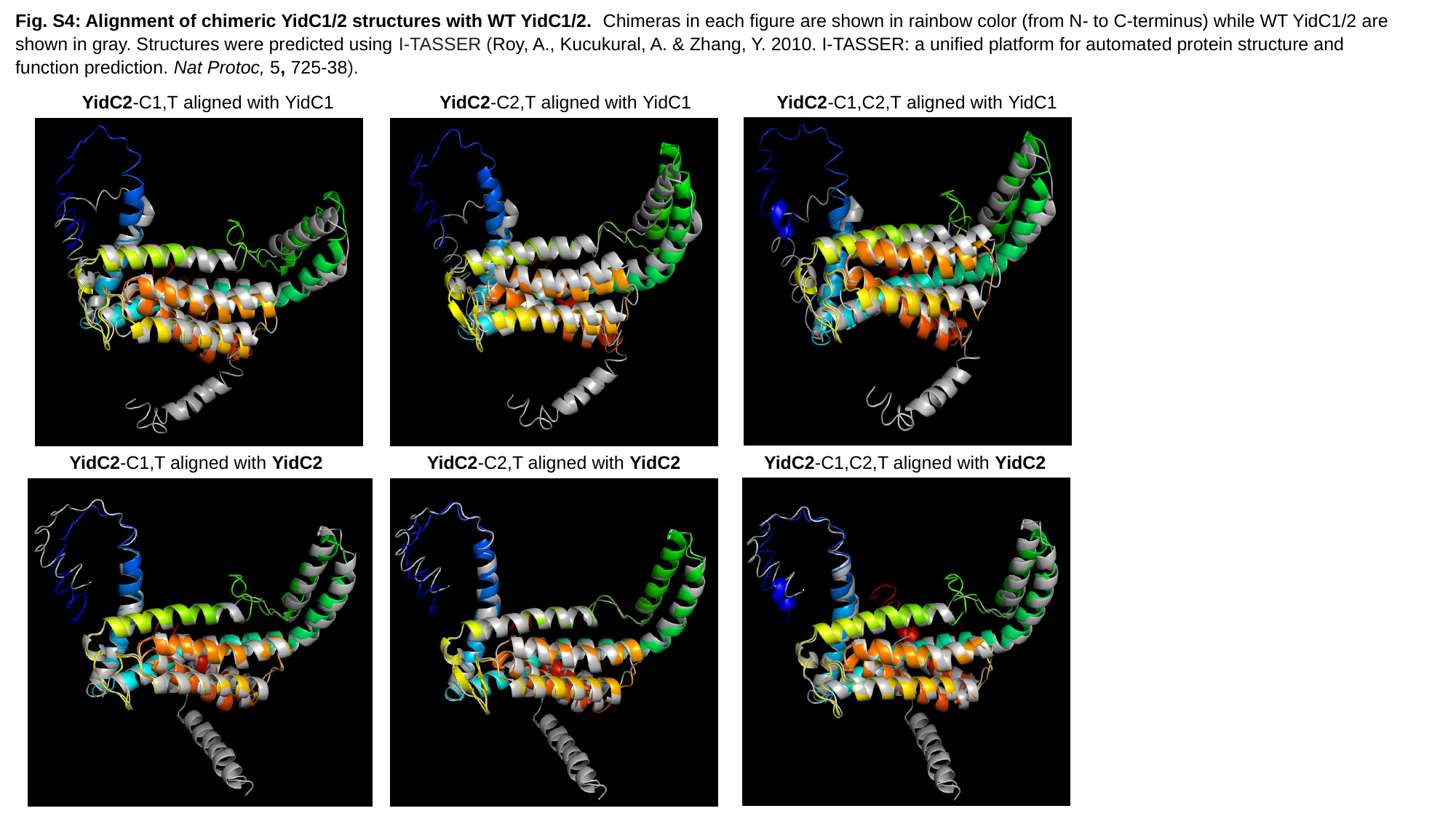

Fig. S4: Alignment of chimeric YidC1/2 structures with WT YidC1/2. Chimeras in each figure are shown in rainbow color (from N- to C-terminus) while WT YidC1/2 are shown in gray. Structures were predicted using I-TASSER (Roy, A., Kucukural, A. & Zhang, Y. 2010. I-TASSER: a unified platform for automated protein structure and function prediction. Nat Protoc, 5, 725-38).
YidC2-C1,T aligned with YidC1
YidC2-C2,T aligned with YidC1
YidC2-C1,C2,T aligned with YidC1
YidC2-C1,T aligned with YidC2
YidC2-C2,T aligned with YidC2
YidC2-C1,C2,T aligned with YidC2

## Slide 10
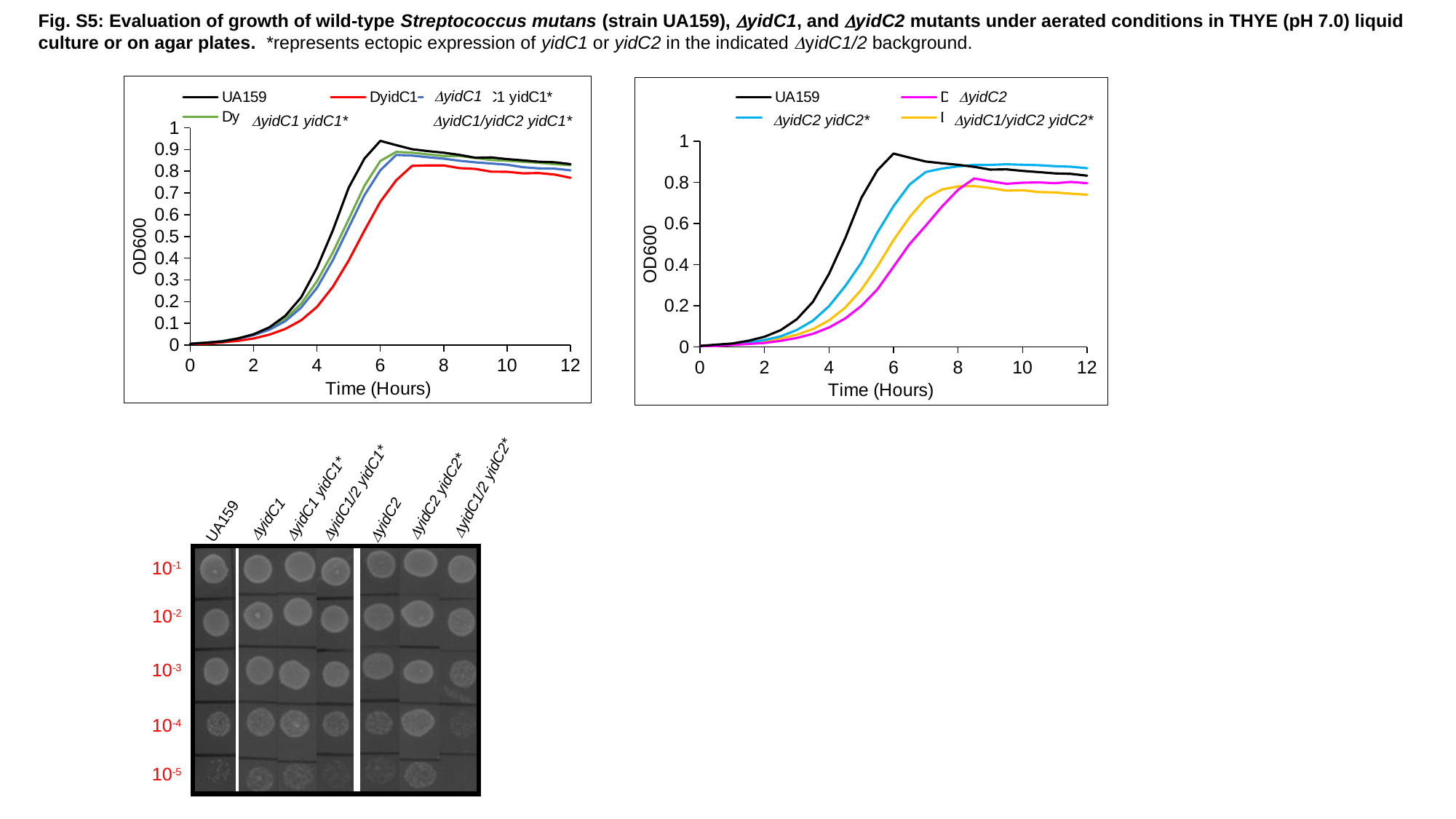

Fig. S5: Evaluation of growth of wild-type Streptococcus mutans (strain UA159), DyidC1, and DyidC2 mutants under aerated conditions in THYE (pH 7.0) liquid culture or on agar plates. *represents ectopic expression of yidC1 or yidC2 in the indicated DyidC1/2 background.
### Chart
| Category | UA159 | DyidC1 | DyidC1 yidC1* | DyidC1/2 yidC1* |
|---|---|---|---|---|
### Chart
| Category | UA159 | DyidC2 | DyidC2 yidC2* | DyidC1/2 yidC2* |
|---|---|---|---|---|DyidC1
DyidC2
DyidC2 yidC2*
DyidC1/yidC2 yidC2*
DyidC1/yidC2 yidC1*
DyidC1 yidC1*
DyidC1/2 yidC2*
DyidC1/2 yidC1*
DyidC1 yidC1*
DyidC2 yidC2*
DyidC1
DyidC2
UA159
10-1
10-2
10-3
10-4
10-5

## Slide 11
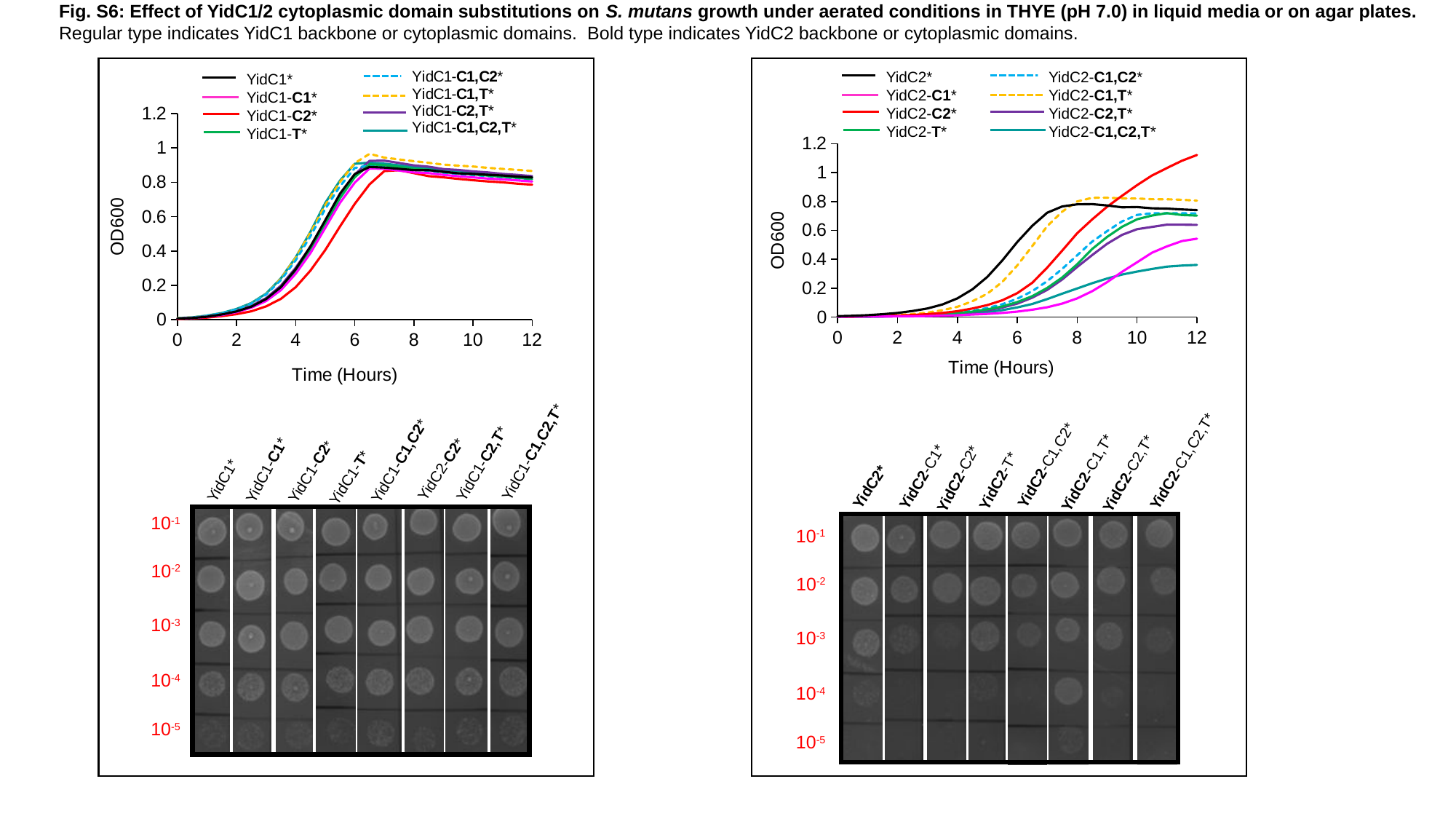

Fig. S6: Effect of YidC1/2 cytoplasmic domain substitutions on S. mutans growth under aerated conditions in THYE (pH 7.0) in liquid media or on agar plates.
Regular type indicates YidC1 backbone or cytoplasmic domains. Bold type indicates YidC2 backbone or cytoplasmic domains.
### Chart
| Category | YidC2 | YidC2-C1 | YidC2-C2 | YidC2-T | YidC2-C1,C2 | YidC2-C1,T | YidC2-C2,T | YidC2-C1,C2,T |
|---|---|---|---|---|---|---|---|---|
YidC2*
YidC2-C1*
YidC2-C2*
YidC2-T*
YidC2-C1,C2*
YidC2-C1,T*
YidC2-C2,T*
YidC2-C1,C2,T*
YidC1*
YidC1-C1*
YidC1-C2*
YidC1-T*
YidC1-C1,C2,T*
YidC1-C1,C2*
YidC2-C1,C2,T*
YidC1-C2,T*
YidC2-C1,C2*
YidC2-C2*
YidC1-C1*
YidC1-C2*
YidC1-T*
YidC2-C2*
YidC2-C1,T*
YidC2-C2,T*
YidC2-T*
YidC1*
YidC2-C1*
YidC2*
10-1
10-2
10-3
10-4
10-5
10-1
10-2
10-3
10-4
10-5
### Chart
| Category | YidC1 | YidC1-C1 | YidC1-C2 | YidC1-T | YidC1-C1,C2 | YidC1-C1,T | YidC1-C2,T | YidC1-C1,C2,T |
|---|---|---|---|---|---|---|---|---|

## Slide 12
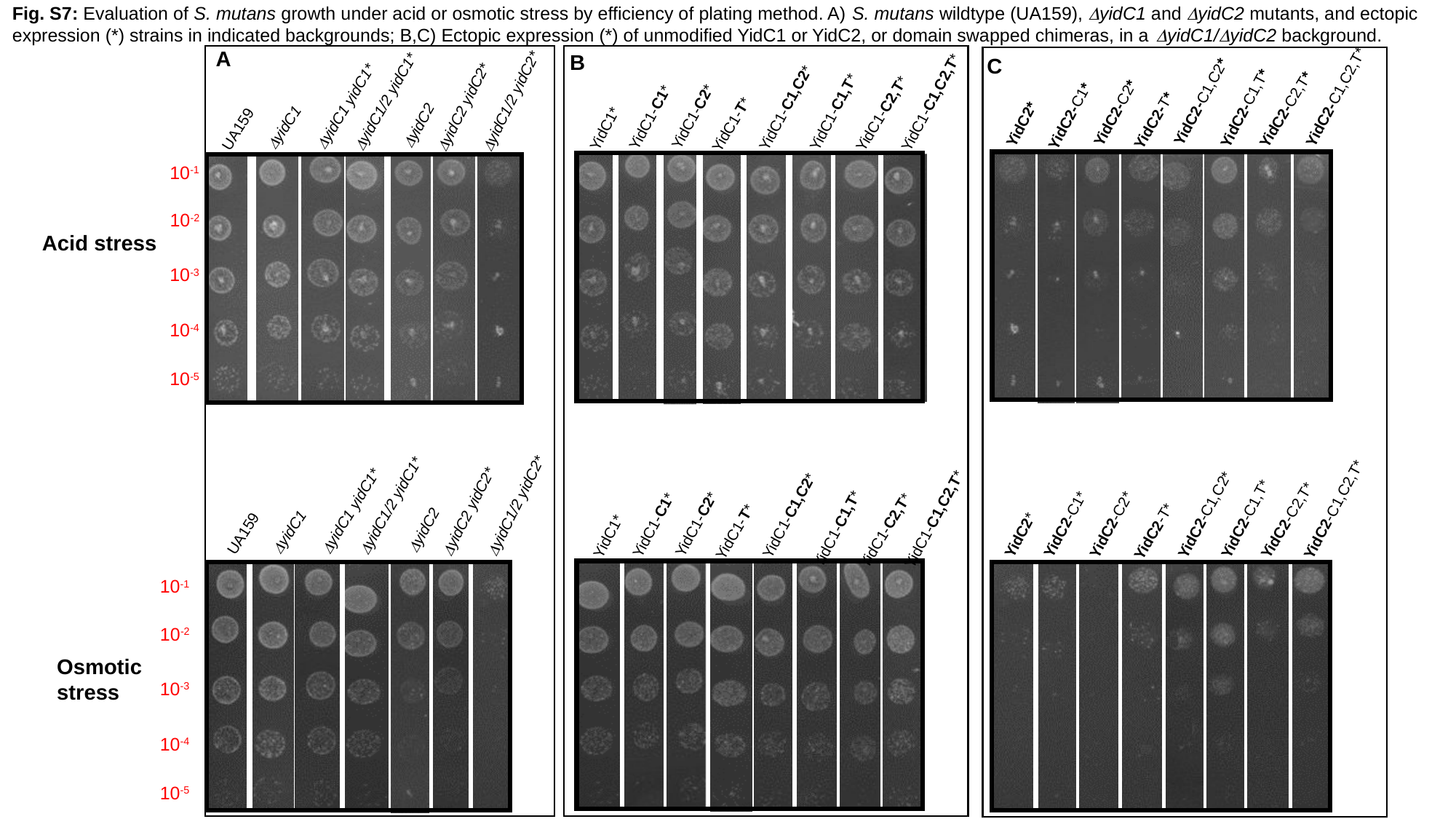

Fig. S7: Evaluation of S. mutans growth under acid or osmotic stress by efficiency of plating method. A) S. mutans wildtype (UA159), DyidC1 and DyidC2 mutants, and ectopic expression (*) strains in indicated backgrounds; B,C) Ectopic expression (*) of unmodified YidC1 or YidC2, or domain swapped chimeras, in a DyidC1/DyidC2 background.
A
B
C
DyidC1/2 yidC2*
YidC2-C1,C2*
DyidC1/2 yidC1*
YidC1-C1,C2*
YidC2-C1,C2,T*
DyidC2 yidC2*
YidC1-C1,C2,T*
DyidC1 yidC1*
YidC2-C1,T*
YidC2-C2,T*
YidC1-C1,T*
YidC2-C2*
YidC1-C2,T*
YidC2-T*
YidC1-C2*
YidC2-C1*
YidC1-C1*
YidC1-T*
DyidC2
YidC2*
DyidC1
UA159
YidC1*
10-1
10-2
10-3
10-4
10-5
Acid stress
DyidC1/2 yidC2*
DyidC1/2 yidC1*
YidC2-C1,C2*
YidC1-C1,C2*
DyidC2 yidC2*
DyidC1 yidC1*
YidC2-C1,C2,T*
YidC1-C1,C2,T*
YidC2-C1,T*
YidC2-C2,T*
YidC1-C2*
YidC2-C1*
YidC1-C1*
YidC2-C2*
DyidC2
YidC1-T*
YidC2-T*
DyidC1
UA159
YidC1-C1,T*
YidC1-C2,T*
YidC2*
YidC1*
10-1
10-2
10-3
10-4
10-5
Osmotic stress

## Slide 13
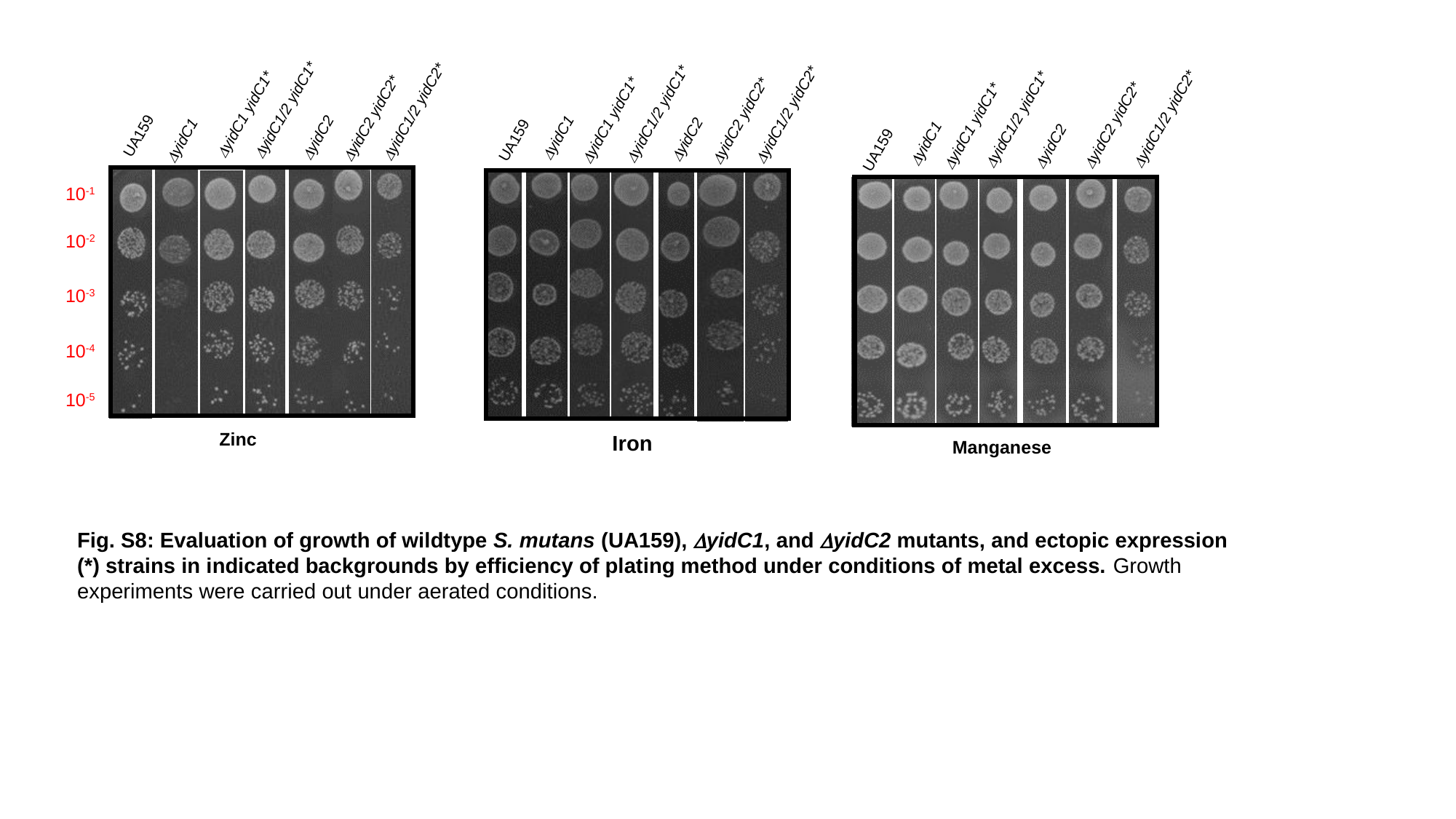

DyidC1/2 yidC1*
DyidC1 yidC1*
DyidC2 yidC2*
DyidC1
DyidC2
UA159
Manganese
DyidC1/2 yidC1*
DyidC1/2 yidC2*
DyidC1/2 yidC1*
DyidC1/2 yidC2*
DyidC2 yidC2*
DyidC2 yidC2*
DyidC1 yidC1*
DyidC1/2 yidC2*
DyidC1 yidC1*
UA159
DyidC1
DyidC2
DyidC2
UA159
DyidC1
10-1
10-2
10-3
10-4
10-5
Zinc
Iron
Fig. S8: Evaluation of growth of wildtype S. mutans (UA159), DyidC1, and DyidC2 mutants, and ectopic expression (*) strains in indicated backgrounds by efficiency of plating method under conditions of metal excess. Growth experiments were carried out under aerated conditions.

## Slide 14
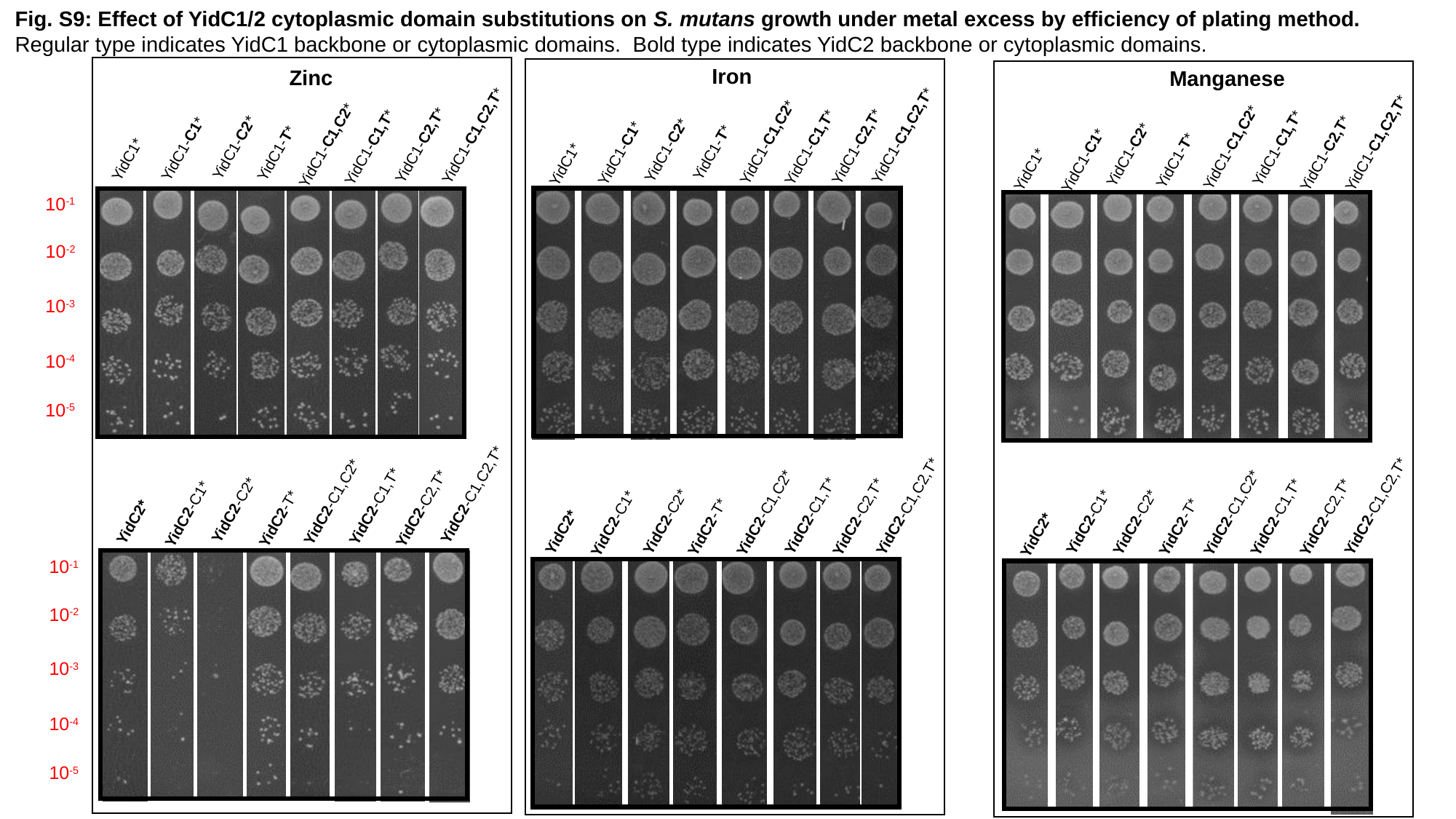

Fig. S9: Effect of YidC1/2 cytoplasmic domain substitutions on S. mutans growth under metal excess by efficiency of plating method.
Regular type indicates YidC1 backbone or cytoplasmic domains. Bold type indicates YidC2 backbone or cytoplasmic domains.
Iron
Zinc
Manganese
YidC1-C1,C2*
YidC1-C1,C2,T*
YidC1-C1,C2,T*
YidC1-C1,C2*
YidC1-C1,C2*
YidC1-C1,C2,T*
YidC1-C2,T*
YidC1-C2,T*
YidC1-C2*
YidC1-C1,T*
YidC1-C1,T*
YidC1-T*
YidC1-C1,T*
YidC1-C1*
YidC1-T*
YidC1-C2*
YidC1-C1*
YidC1-C2,T*
YidC1-C2*
YidC1-T*
YidC1*
YidC1-C1*
YidC1*
YidC1*
10-1
10-2
10-3
10-4
10-5
YidC2-C1,C2,T*
YidC2-C1,C2*
YidC2-C1,C2,T*
YidC2-C1,C2,T*
YidC2-C1,T*
YidC2-C1,C2*
YidC2-C2,T*
YidC2-C1,C2*
YidC2-C2*
YidC2-C1*
YidC2-T*
YidC2-C1,T*
YidC2-C2,T*
YidC2-C1,T*
YidC2-C2,T*
YidC2*
YidC2-C2*
YidC2-C1*
YidC2-C2*
YidC2-T*
YidC2-T*
YidC2-C1*
YidC2*
YidC2*
10-1
10-2
10-3
10-4
10-5
