# Supplementary material for: The Cytoplasmic Domains of Streptococcus mutans Membrane Protein Insertases YidC1 and YidC2 Confer Unique Structural and Functional Attributes to Each Paralog
Source: Front Microbiol. 2021 Nov 2;12:760873. doi: 10.3389/fmicb.2021.760873 (PMC8595059; doi:10.3389/fmicb.2021.760873)
Supplement: Supplementary file 2 [file Table_1.DOCX]

**Table S1. List of bacterial strains and plasmids used in this study.**

| **Strains** | **Genotype/description** | **Source/reference** |
| --- | --- | --- |
| ***Streptococcus mutans*** | | |
| UA159 | Wild-type (serotype c) | Ajdic et al., 2002 |
| SM2001 | UA159 *ΔyidC1::*aad9; Spc^R^ | This work |
| SM1081 | UA159 *ΔyidC2::aphA-3*; Kan^R^ | This work |
| SM2140 | UA159 *gtfA::erm*; Erm^R^ | This work |
| SM2253 | UA159 *ΔyidC1::aad9 gtfA::erm*; Spc^R^, Erm^R^ | This work |
| SM2143 | UA159 *ΔyidC2::aphA3 gtfA::ermB*; Kan^R^, Erm^R^ | This work |
| SM2035 | UA159 *ΔyidC1::aad9 gtfA::yidC1*; Spc^R^, Erm^R^ | This work |
| SM1106 | UA159 *ΔyidC2::aphA-3 gtfA::yidC2*; Kan^R^, Erm^R^ | This work |
| SM2107 | UA159 *ΔyidC1::aad9 ΔyidC2::aphA-3 gtfA::yidC1*; Spc^R^, Kan^R^, Erm^R^ | This work |
| SM2146 | UA159 *ΔyidC1::aad9 ΔyidC2::aphA-3 gtfA::yidC1(yidC2-C1 loop)*(Hyb); Spc^R^, Kan^R^, Erm^R^ | This work |
| SM2112 | UA159 *ΔyidC1::aad9 ΔyidC2::aphA-3 gtfA::yidC1(yidC2-C2 loop)*(Hyb); Spc^R^, Kan^R^, Erm^R^ | This work |
| SM2117 | UA159 *ΔyidC1::aad9 ΔyidC2::aphA-3 gtfA::yidC1(yidC2-tail)*(Hyb); Spc^R^, Kan^R^, Erm^R^ | This work |
| SM2245 | UA159 *ΔyidC1::aad9 ΔyidC2::aphA-3 gtfA::yidC1(yidC2-C1 loop, C2 loop)*(Hyb); Spc^R^, Kan^R^, Erm^R^ | This work |
| SM2208 | UA159 *ΔyidC1::aad9 ΔyidC2::aphA-3 gtfA::yidC1(yidC2-C1 loop, tail)*(Hyb); Spc^R^, Kan^R^, Erm^R^ | This work |
| SM2150 | UA159 *ΔyidC1::aad9 ΔyidC2::aphA-3 gtfA::yidC1(yidC2-C2 loop, tail)*(Hyb); Spc^R^, Kan^R^, Erm^R^ | This work |
| SM2123 | UA159 *ΔyidC1::aad9 ΔyidC2::aphA-3 gtfA::yidC1(yidC2-C1 loop, C2 loop, tail)*(Hyb); Spc^R^, Kan^R^, Erm^R^ | This work |
| SM2231 | UA159 *yidC1::aad9 ΔyidC2::aphA-3 gtfA::yidC2*; Spc^R^, Kan^R^, Erm^R^ | This work |
| SM2228 | UA159 *ΔyidC1::aad9 ΔyidC2::aphA-3 gtfA::yidC1(yidC1-C1 loop)*(Hyb); Spc^R^, Kan^R^, Erm^R^ | This work |
| SM2077 | UA159 *ΔyidC1::aad9 ΔyidC2::aphA-3 gtfA::yidC1(yidC1-C2 loop)*(Hyb); Spc^R^, Kan^R^, Erm^R^ | This work |
| SM2233 | UA159 *ΔyidC1::aad9 ΔyidC2::aphA-3 gtfA::yidC1(yidC1-tail)*(Hyb); Spc^R^, Kan^R^, Erm^R^ | This work |
| SM2078 | UA159 *ΔyidC1::aad9 ΔyidC2::aphA3 gtfA::yidC1(yidC1-C1 loop, C2 loop)*(Hyb); Spc^R^, Kan^R^, Erm^R^ | This work |
| SM2083 | UA159 *ΔyidC1::aad9 ΔyidC2::aphA-3 gtfA::yidC1(yidC1-C1 loop, tail)*(Hyb); Spc^R^, Kan^R^, Erm^R^ | This work |
| SM2087 | UA159 *ΔyidC1::aad9 ΔyidC2::aphA-3 gtfA::yidC1(yidC1-C2 loop, tail)*(Hyb); Spc^R^, Kan^R^, Erm^R^ | This work |
| SM2089 | UA159 *ΔyidC1::aad9 ΔyidC2::aphA-3 gtfA::yidC1(yidC1-C1 loop, C2 loop, tail)*(Hyb); Spc^R^, Kan^R^, Erm^R^ | This work |
| SM2297 | UA159 *ΔyidC1::aad9 ΔyidC2::aphA-3 gtfA::yidC1^K91A^*; Spc^R^, Kan^R^, Erm^R^ | This work |
| SM2303 | UA159 *ΔyidC1::aad9 ΔyidC2::aphA-3 gtfA::yidC1^E190A^*; Spc^R^, Kan^R^, Erm^R^ | This work |
| SM2309 | UA159 *ΔyidC1::aad9 ΔyidC2::aphA-3 gtfA::yidC2^E92A^*; Spc^R^, Kan^R^, Erm^R^ | This work |
| SM2312 | UA159 *ΔyidC1::aad9 ΔyidC2::aphA-3 gtfA::yidC2^K253A^*; Spc^R^, Kan^R^, Erm^R^ | This work |
| ***Escherichia coli*** | | |
| C2987 | *fhuA2 Δ(argF-lacZ)U169 phoA glnV44 f80Δ(lacZ)M15 gyrA96 recA1 relA1 endA1 thi-1 hsdR17* | NEB |
| Plasmids | | |
| pET15b | Expression vector, Amp^R^ | Novagen |
| pBGE | Integration vector for integration at *gtfA* locus in  *S. mutans* | Zeng and Burne, 2010 |
| pCR2.1-ΔyidC1-sp | pCR2.1 with *ΔyidC1::sp* for allelic replacement of *yidC1*. | Palmer et al., 2012 |
| pAH342 | pBAD vector with *ΔyidC2::aphA3* for allelic replacement of *yidC2*. | Hasona et al., 2005 |

**Table S2: Details of the relevant amino acid residues in the chimeric YidC1/2 variants.** YidC1 backbone and cytoplasmic domains are indicated in regular text while YidC2-derived segments are indicated in bold type.

| **Protein** | **Description of chimeric protein** | **Amino acids replacements** |
| --- | --- | --- |
| YidC1 | Wild-type YidC1 (1-271 aa) | None |
| YidC1-**C1** | YidC1 containing cytoplasmic loop C1 of YidC2. | Amino acids 73-126 (56 aa) of YidC1 replaced with amino acids 76-131 of YidC2 (56 aa). |
| YidC1-**C2** | Chimeric YidC1 containing cytoplasmic loop C2 of YidC2. | Amino acids 183-196 (14 aa) of YidC1 replaced with amino acids 195-211 of YidC2 (17 aa). |
| YidC1-**T** | Chimeric YidC1 containing C-terminal tail of YidC2. | Amino acids 233-271 (38 aa) of YidC1 replaced with amino acids 247-310 (63 aa) of YidC2. |
| YidC1-**C1,C2** | Chimeric YidC1 containing cytoplasmic loop 1 and 2 of YidC2. | Amino acids 73-126 (56 aa) and 183-196 (14 aa) of YidC1 replaced with aa 76-131 (56 aa) and 195-211 (17 aa), respectively. |
| YidC1-**C1,T** | Chimeric YidC1 containing cytoplasmic loop1 and C-terminal tail of YidC2. | Amino acids 73-126 (56 aa) and 233-271 (38 aa) of YidC1 replaced with aa 76-131 (56 aa) and 247-310 (64 aa), respectively. |
| YidC1-**C2,T** | Chimeric YidC1 containing cytoplasmic loop 2 and C-terminal tail of YidC2. | Amino acids 183 to 196 (14 aa) and 233-271 (38 aa) of YidC1 replaced with aa 195-211 (17 aa) and 247-310 (64 aa), respectively. |
| YidC1-**C1,C2,T** | Chimeric YidC1 containing cytoplasmic loop1, 2, and C-terminal tail of YidC2. | Amino acids 73-126 (56 aa), 183-196 (14 aa), and 233-271 (47 aa) of YidC1 replaced with amino acids 76-131 (56 aa), 195-211 (17 aa), and 248-310 (64 aa) of YidC2, respectively. |
| **YidC2** | Wild-type YidC2 (1-310 aa) | None |
| **YidC2**-C1 | Chimeric YidC2 containing cytoplasmic loop C1 of YidC1. | Amino acids 76-131 (56 aa) of YidC2 replaced with amino acids 73-126 (56 aa) of YidC1. |
| **YidC2**-C2 | Chimeric YidC2 containing cytoplasmic loop C2 of YidC1. | Amino acids 195-211 (17 aa) of YidC2 replaced with amino acids 183-196 (14 aa) of YidC1. |
| **YidC2**-T | Chimeric YidC2 containing C-terminal tail of YidC1. | Amino acids 247-310 (64 aa) of YidC2 replaced with 233-271 (38 aa) of YidC1. |
| **YidC2**-C1,C2 | Chimeric YidC2 containing cytoplasmic loop 1 and 2 of YidC1. | Amino acids 76-131 (56 aa) and 195-211 (17 aa) of YidC2 replaced with amino acids 73-126 (56 aa) and 183-196 (14 aa) of YidC1, respectively. |
| **YidC2**-C1,T | Chimeric YidC2 containing cytoplasmic loop1 and C-terminal tail of YidC1. | Amino acids 76-131 (56 aa) and 247-310 (64 aa) of YidC2 replaced with amino acids 73-126 (56 aa) and 233-271 (38 aa) of YidC1, respectively. |
| **YidC2**-C2,T | Chimeric YidC2 containing cytoplasmic loop 2 and C-terminal tail of YidC1. | Amino acids 195-211 (17 aa) and 247-310 (64 aa) of YidC2 replaced with 183-196 (14 aa) and 233-271 (38 aa) of YidC2, respectively. |
| **YidC2**-C1,C2,T | Chimeric YidC1 containing cytoplasmic loop1, 2, and C-terminal tail of YidC2. | Amino acids 76-131 (56 aa), 195-211 (17 aa), and 247-310 (64 aa) of YidC2 replaced with 73-126 (56 aa), 183-196 (14 aa), and 233-271 (38 aa) of YidC1, respectively. |

**Table S3: List of primers used in this work.** Uppercase letters in primer sequences represent sequences corresponding to respective genes in *S. mutans* strain UA159 while lowercase letters represent non-homologous sequences.

| **Name** | **Sequence (5’🡪3’)** | **Restriction enzyme** | **Direction** | **Location** | **Description** |
| --- | --- | --- | --- | --- | --- |
| SM1 | ctggtgccgcgcggcagccacgcggcagccatatgGTGAAAAAGAAATATAGAATTATTGG | NdeI | Fwd | +1 to +26 bp of *yidC1*. | PCR amplification of DNA fragment corresponding to 1-72 aa of YidC1. |
| SM2 | atggcaagatCAGTGGTAAAAGAATGGTTC | None | Rev | +197 to +216 bp of *yidC1*. |  |
| SM3 | tttaccactgATCTTGCCATTAGGTCTTAGTTCAAG | None | Rev | +376 to +393 bp of *yidC2*. | PCR amplification of C1 loop of *yidC2* (76-131 aa) to construct chimeric YidC1-**C1**. |
| SM4 | gaggaaaaagGTAATGACGCTGCGCCTG | None | Rev | +376 to +393 bp of *yidC2*. |  |
| SM5 | gcgtcattacCTTTTTCCTCTTCTTATTCAG | None | Fwd | +379 to +399 bp of *yidC1* | PCR amplification of DNA fragment corresponding to 127-271 aa of YidC1. |
| SM6 | tcgggctttgttagcagccgggatcctcgaTTATTTTCTCTTTTTATGTGCTTTC | BamHI | Rev | +792 to +816 bp of *yidC1*. |  |
| SM7 | ctggtgccgcgcggcagccatatgcgcggcagccatatgGTGAAAAAGAAATATAGAATTATTGG | NdeI | Fwd | +1 to +26 bp of *yidC1*. | PCR amplification of DNA fragment corresponding to 1-182 aa of YidC1. |
| SM8 | ggaacacttagcgttgaCCAAGAAGATAAGAAAGTAAAAATAG | None | Rev | +521 to +546 bp of *yidC1*. |  |
| SM9 | atcttcttggtcaacgctaagtgttccagaagcacaaagacagcaaacaagaaatatgACGATGAACATTATCTTACC | None | Fwd | +585 to +633 bp of *yidC2*. | PCR amplification of C2 loop of YidC2 (195-211 aa) with 197-271 aa of YidC1. |
| SM10 | tcgggctttgttagcagccggatccagccggatccTTATTTTCTCTTTTTATGTGCTTTC | BamHI | Rev | +792 to +816 bp. |  |
| SM11 | ctggtgccgcgcggcagccatatgcgcggcagccatatgGTGAAAAAAATTTACAAGCGTC | NdeI | Fwd | +1 to +22 bases of *yidC2*. | PCR amplification of DNA fragment corresponding to 1-75 aa of YidC2. |
| SM12 | gcaagttaaaGAGAAGACGGACAATAAC | None | Rev | +208 to +225 bp of *yidC2*. |  |
| SM13 | ccgtcttctcTTTAACTTGCAATTGAAATCTGG | None | Fwd | +217 to +239 bp of *yidC1*. | PCR amplification of C1 loop of YidC1 (73-126 aa). |
| SM14 | tgctcataccGCTGGCATAGGGATTGAC | None | Rev | +361 to +378 bp of *yidC1*. |  |
| SM15 | ctatgccagcGGTATGAGCATGTTTGGTG | None | Fwd | +394 to +412 bp of *yidC2*. | PCR amplification of DNA fragment corresponding to 132-310 aa of YidC2. |
| SM16 | tcgggctttgttagcagccggatccagccggatccTTATTGCTTATGGTGACG | BamHI | Rev | +916 to +933 bp of *yidC2*. |  |
| SM35 | ctggtgccgcgcggcagccatatgcgcggcagccatatgGTGAAAAAAATTTACAAGCGTC | NdeI | Fwd | +1 to +22 bp of *yidC2* | PCR amplification of DNA fragment corresponding to 1-194 aa of YidC2. |
| SM36 | gcggccttattagtcagCACCCAAGACTGAACAAG | None | Rev | +567 to +585 bp of *yidC2* |  |
| SM37 | gtcttgggtgctgactaataaggccgccaaagaacgaaatggtatgatgataATGTTTATGATGCCAATTATG | None | Fwd | +633 to +654 bp of *yidC2*. | PCR amplification of DNA fragment corresponding to 212-310 aa of YidC2 with C1 loop of YidC1. |
| SM38 | tcgggctttgttagcagccggatccagccggatccTTATTGCTTATGGTGACG | BamHI | Rev | +916 to +933 bp of *yidC2*. |  |
| SM200 | ccttatttaggcactctaga*ttaa*ATGAAAAAGAAATATAGAATTATTGGATTAG | XbaI | Fwd | +2 to +30 bp of *yidC1*. | PCR amplification of YidC1 variants for subcloning in pBGE. |
| SM82 | gcttccgtttgtacaTTATTTTCTCTTTTTATGTGCTTTCTTTTTAG | BsrGI | Rev | +785 to +816 bp of *yidC1*. |  |
| SM201 | ccttatttaggcactctagattaaATGAAAAAAATTTACAAGCGTCTT | XbaI | Fwd | +2 to +24 bp of *yidC2*. | PCR amplification of YidC2 variants for subcloning in pBGE |
| SM84 | gcttccgtttgtacaTTATTGCTTATGGTGACGCTG | BsrGI | Rev | +913 to +933 bp of *yidC2*. |  |
| SM221 | GTATCGGGTGCGGAAATCTT | None | Fwd | +655 to +674 bp of *gtfA* | Sequencing/verification primers for integration at *gtfA* locus. |
| SM222 | GACATTGGCACCGACCTTAT | None | Rev | +926 to +945 bp of *gtfA* |  |
| SM282 | ctggtgccgcgcggcagccaGTGAAAAAGAAATATAGAATTATTGG | None | Fwd | +1 to +26 bp of *yidC*1 | PCR amplification of DNA fragment corresponding to 1-233 aa of YidC1. |
| SM283 | ggtttgtgatCAGAATCTGGAAGACCTG | None | Rev | +682 to +699 bp of *yidC1* |  |
| SM284 | ccagattctgATCACAAACCATATCATTAAAC | None | Fwd | +739 to +760 bp of *yidC2* | PCR amplification of DNA fragment corresponding to 247-310 aa of YidC2. |
| SM285 | tcgggctttgttagcagccgTTATTGCTTATGGTGACG | None | Rev | +916 to +933 bp of *yidC2* |  |
| SM286 | ctggtgccgcgcggcagccaGTGAAAAAAATTTACAAGCGTC | None | Fwd | +1 to +22 bp of *yidC2* | PCR amplification of DNA fragment corresponding to 1-246 aa of YidC2. |
| SM287 | tatttaacaaCAATTGTTGGATGAGACC | None | Rev | +721 to +738 bp of *yidC2* |  |
| SM288 | ccaacaattgTTGTTAAATAATCCTTTTAAAATTATTGC | None | Fwd | +700 to +728 bp of *yidC1* | PCR amplification of DNA fragment corresponding to 233-271 aa of YidC1. |
| SM289 | tcgggctttgttagcagccgTTATTTTCTCTTTTTATGTGCTTTC | None | Rev | +792 to +816 bp of *yidC1* |  |
| SM463 | GAGCTGgctGCTTTACAGACTAAATATCC | None | Fwd | +265 to +292 bp of *yidC1* (K91A) | PCR-based mutagenesis of YidC1 to YidC1^K91A^ |
| SM464 | GTAAAGCAGCCAGCTCAGGCTGAAG | None | Rev | +256 to +280 bp of *yidC1* (K91A) |  |
| SM465 | CCAAAGctCGAAATGGTATGATGATAAC | None | Fwd | +563 to +590 bp of *yidC1* (E190A) | PCR-based mutagenesis of YidC1 to YidC1^E190A^ |
| SM466 | CATTTCGagCTTTGGCGG | None | Rev | +560 to +577 bp of *yidC1* (E190A) |  |
| SM467 | TATCATTgctCCAAAATTGCG  GAAG | None | Fwd | +750 to +774 bp of *yidC2* (K253A) | PCR-based mutagenesis of YidC2 to YidC2^K253^*^.^* |
| SM468 | ATTTTGGagcAATGATATGGTTTGTGATC | None | Rev | +738 to +766 bp of *yidC2* (K253A) |  |
| SM135 | TTACCAATCTgctAAAATGGCTTATC | None | Fwd | +264 to +289 bp of *yidC2* (E92A) | PCR-based mutagenesis of YidC2 to YidC2^E92A^ |
| SM136 | GTCATCTTTCGTACTTGAC | None | Rev | +245 to +263 bp of *yidC2* (E92A) |  |

**Table S4: Mean generation times of *S. mutans* WT and mutant strains grown in THYE media under non-stress (THYE, pH 7.0, anaerobic) or indicated stress conditions.** Mean generation times (min) ±sd were calculated based on triplicate OD_600_ values (during exponential phase) measured in a Bioscreen C 100-well plate from at least three independent experiments. YidC1 backbone and cytoplasmic domains are indicated in regular text while YidC2-derived segments are indicated in bold type. *indicates ectopic expression from the *gtfA* locus in a double *ΔyidC1/ΔyidC2* background unless single *ΔyidC* or *ΔyidC2* backgrounds are indicated. NG indicates no growth.

| **Strains** | **THYE**  **(pH 7, anaerobic)** | **THYE**  **(pH 7, air)** | **THYE**  **(pH 5)** | **THYE**  **(3% NaCl)** | **THYE**  **(5 mM FeSO_4_)** | **THYE**  **(5 mM MnCl_2_)** | **THYE**  **(2.5 mM ZnCl_2_)** |
| --- | --- | --- | --- | --- | --- | --- | --- |
| UA159 | 54.78 ± 3.51 | 42.03 ± 1.39 | 168.8 ± 12.2 | 99.6 ± 6.23 | 44.2 ± 4.9 | 50.3 ± 2.4 | 60.68 ± 12.6 |
| UA159 *gtfA::ermB* | 50.76 ± 6.78 | 39 ± 2.96 | 153.4 ± 14 | 103.6 ± 6 | 42.65 ± 1.06 | 47.04 ± 0.33 | 59.6 ± 8.8 |
| *ΔyidC1* | 52.95 ± 3.65 | 45.5 ± 3.5 | 146.2 ± 7.5 | 98.6 ± 5.4 | 40.7 ± 5.3 | 53.2 ± 1.13 | NG |
| *ΔyidC1 gtfA::ermB* | 49.65 ± 1.9 | 45.3 ± 2.6 | 205 ± 13.4 | 117.6 ± 8.5 | 41.3 ± 0.2 | 52.2 ± 2.5 | NG |
| *ΔyidC2* | 67.4 ± 5 | 51.5 ± 2.4 | NG | 133.6 ± 2.6 | 48.5 ± 2.2 | 64.1 ± 4.4 | 70.6 ± 6.4 |
| *ΔyidC2* *gtfA::ermB* | 65.4 ± 4 | 53.45 ± 3.57 | NG | 128.6 ± 6.4 | 51.4 ± 1.06 | 63.6 ± 3.4 | 69.6 ± 9.3 |
| *ΔyidC1* *yidC1** | 53.5 ± 5.9 | 46.4 ± 1.9 | 257 ± 12.8 | 106.6 ± 3.5 | 47.9 ± 0.9 | 53.2 ± 1.06 | 69.9 ± 12 |
| *ΔyidC2* *yidC2** | 55.67 ± 2.95 | 44.13 ± 0.3 | 263 ± 6.35 | 117.8 ± 5.37 | 50.3 ± 3.7 | 63.6 ± 9.3 | 70.8 ± 7.57 |
| YidC1* | 53.8 ± 5.5 | 45.2 ± 1.8 | 260 ± 19 | 104 ± 2.8 | 47.4 ± 2.12 | 50.8 ± 0.7 | 69.9 ± 11.4 |
| YidC2* | 66.1 ± 5.2 | 56.4 ± 1.1 | 312 ± 32 | 142.6 ± 1.73 | 64.4 ± 6.1 | 110 ± 12 | 83.1 ± 7.1 |
| YidC1-**C1*** | 52.6 ± 3.45 | 42.8 ± 1.3 | 228 ± 9.8 | 105.4 ± 3.6 | 50.2 ± 2.2 | 48.3 ± 1.3 | 68.4 ± 9.8 |
| YidC1-**C2*** | 54.4 ± 4.5 | 46.7 ± 1.8 | 182.4 ± 11 | 112 ± 7.7 | 48.2 ± 0.65 | 52.8 ± 0 | 77.4 ± 10 |
| YidC1-**T*** | 52.7 ± 2.1 | 46.8 ± 3.7 | 222.8 ± 9.5 | 103.3 ± 3.6 | 50.2 ± 2.2 | 51.4 ± 0.04 | 76.2 ± 9.5 |
| YidC1-**C1,C2*** | 49.9 ± 4.9 | 44.3 ± 2.8 | 225 ± 21 | 111.4 ± 4.4 | 49.5 ± 2.9 | 50.2 ± 0.33 | 70.5 ± 9.7 |
| YidC1-**C1,T*** | 55.8 ± 5.9 | 47.07 ± 2.58 | 191 ± 7.06 | 106.8 ± 2.74 | 43.9 ± 3.4 | 55.5 ± 6.36 | 84.5 ± 12 |
| YidC1-**C2,T*** | 55.2 ± 6.1 | 48.5 ± 2.3 | 236.7 ± 12.7 | 106.1 ± 1.8 | 51.5 ± 2.4 | 48.8 ± 0.2 | 74.9 ± 18.2 |
| YidC1-**C1,C2,T*** | 59.5 ± 6.2 | 45.2 ± 1.9 | 189.1 ± 8.4 | 106 ± 6.1 | 50.3 ± 5.6 | 47.7 ± 0.7 | 73.5 ±13 |
| **YidC2**-C1* | 82.2 ± 4.2 | 73.3 ± 3.2 | Trace growth | 213.4 ± 8 | 47.8 ± 3.6 | 68.9 ± 6.8 | 87.4 ± 11.3 |
| **YidC2**-C2* | 70.6 ± 3.3 | 62.46 ± 3.1 | 291 ± 20 | 199.4 ± 15 | 66 ± 5 | 69.6 ± 6.7 | NG |
| **YidC2**-T* | 68.3 ± 2.2 | 62.4 ± 0 | Trace growth | 165 ± 13 | 62.1 ± 0.4 | 68.4 ± 1.7 | 78.9 ± 5.5 |
| **YidC2**-C1,C2* | 83.4 ± 4.6 | 58.1 ± 2.6 | Trace growth | 167 ± 19 | 58.1 ± 1.43 | 82.5 ± 12 | 95 ± 11 |
| **YidC2**-C1,T* | 47.4 ± 4.2 | 46.8 ± 2.8 | 195.3 ± 7.4 | 115.3 ± 1.2 | 47.7 ± 2.4 | 52.5 ± 2.12 | 118.8 ± 19.5 |
| **YidC2**-C2,T* | 58.6 ± 5.25 | 60.1 ± 4.5 | 279 ± 13 | 119.4 ± 3.3 | 55.6 ± 1.4 | 62.1 ± 1.27 | 73.8 ± 5.9 |
| **YidC2**-C1,C2,T* | 83.5 ± 8.2 | 66.5 ± 3.2 | Trace growth | 146.6 ± 10.2 | 61.7 ± 0.04 | 81.9 ± 4.6 | 82.8 ± 2.54 |

**Table S5:** **Mass spectrometry protein scores of proteins involved in Fe(II), Mn(II), and Zn(II) homeostasis detected in membrane preparations of wild-type *S. mutans* (strain NG8) and deletion mutants lacking one or more protein translocation machinery components.*** Protein scores were calculated from the mean of five replicates.

| **Metal** | **Transporter/regulator** | **Protein Scores (PEP SUM Scores)** | | | | |
| --- | --- | --- | --- | --- | --- | --- |
|  |  | **WT** | ***Δffh*** | ***ΔyidC1*** | ***ΔyidC2*** | ***Δffh/ΔyidC1*** |
| Fe/Mn | SloR | 0 | 0.88 ± 0.84 | 4.58 ± 1.64 | 1 ± 0.03 | 0 |
|  | SloA | 7.94 ± 0.75 | 5.41 ± 1.54 | 7.11 ± 1.48 | 9.04 ± 2.13 | 5.75 ± 1.23 |
|  | SloC | 15.66 ± 4.89 | 15.14 ± 2.04 | 16.12 ± 2.74 | 15.85 ± 2.48 | 10.65 ± 2.86 |
|  | MntH | 4.9 ± 1.5 | 4.75 ± 1.7 | 5.68 ± 0.97 | 4.81 ± 1.5 | 2.16 ± 0.99 |
|  | Dpr | 3.46 ± 1.23 | 2.45 ± 0.92 | 4.05 ± 0.8 | 3.18 ± 1.25 | 1.62 ± 1.25 |
|  | FeoA | 1.06 ± 1.01 | 0 | 0 | 0 | 0.69 ± 0.63 |
|  | FeoB | 3.8 ± 0.49 | 3.27 ± 0.79 | 2.31 ± 0.81 | 3.58 ± 1.09 | 0 |
|  | SMU_998 | 5.56 ± 1.23 | 2.84 ± 2.48 | 6.44 ± 1.25 | 3.8 ± 1.3 | 1.52 ± 1.1 |
| Zn | AdcA | 9.5 ± 2.15 | 9.01 ± 3.35 | 7.25 ± 0.69 | 7.98 ± 0.81 | 5.34 ± 1.4 |
| Cu | CopA | 5.62 ± 0.76 | 4.45 ± 1.77 | 4.86 ± 1.22 | 5.04 ± 1.72 | 1.12 ± 0.77 |
| Other | SMU_2057 | 4.76 ± 2.06 | 0 | 0.97 ± 0.63 | 4 ± 0.7 | 1.96 ± 1.13 |

*Extracted from supplemental data of Mishra et al. (2019) *Molecular Oral Microbiology*. Protein scores represent mean of 5 replicates ± SD.

**Table S6: List of putative salt bridges identified between indicated amino acid residues present in the cytoplasmic domains of SmYidC1 and SmYidC2.**

| **Protein** | **Residues (Cytoplasmic domain)** | **Distance (Å)** |
| --- | --- | --- |
| YidC1 | E89 (C1 loop)-K96 (C1 loop) | 2.7 |
|  | K91 (C1 loop)-E190 (C2 loop) | 2.7 |
|  | D101 (C1 loop)-R102 (C1 loop) | 2.8 |
|  | R107 (C1 loop)-E111 (C1 loop) | 2.7 |
|  | K240 (C-terminal tail)-E244 (C-terminal tail) | 2.7 |
|  | R245 (C-terminal tail)-E249 (C-terminal tail) | 2.8 |
|  | E246 (C-terminal tail)-K259 (C-terminal tail) | 2.7 |
|  | E256 (C-terminal tail)-K263 (C-terminal tail) | 2.7 |
|  | E254 (C-terminal tail)-K268 (C-terminal tail) | 2.9 |
| YidC2 | K98 (C1 loop)-D102 (C1 loop) | 2.7 |
|  | E106 (C1 loop)-K109 (C1 loop) | 2.7 |
|  | R107(C1 loop)-E116 (C1 loop) | 2.7, 2.9 |
|  | R107 (C1 loop)-E123 (C1 loop) | 2.8, 2.9 |
|  | E92 (C1 loop)-K253 (C-terminal tail) | 2.7 |
|  | E202 (C2 loop)-R205 (C2 loop) | 2.7 |
|  | K255 (C-terminal tail)-D287 (C-terminal tail) | 2.7 |
|  | R257 (C-terminal tail)-D261 (C-terminal tail) | 2.7 |
|  | K265 (C-terminal tail)-E261 (C-terminal tail) | 2.8 |
|  | D261 (C-terminal tail)-K265(C-terminal tail) | 2.7 |
